# Lessons from mouse chimaera experiments with a reiterated transgene marker: revised marker criteria and a review of chimaera markers

**DOI:** 10.1007/s11248-015-9883-7

**Published:** 2015-06-06

**Authors:** Margaret A. Keighren, Jean Flockhart, Benjamin A. Hodson, Guan-Yi Shen, James R. Birtley, Antonio Notarnicola-Harwood, John D. West

**Affiliations:** Genes and Development Group, Centre for Integrative Physiology, School of Clinical Sciences, University of Edinburgh, Hugh Robson Building, George Square, Edinburgh, EH8 9XD UK; Medical and Developmental Genetics Section, MRC Human Genetics Unit, MRC IGMM, University of Edinburgh, Western General Hospital, Crewe Road, Edinburgh, EH4 2XU UK; Pathology Department, University of Massachusetts Medical School, Worcester, MA 01605 USA; NIC International College in Japan, 5-9-16 Shinjuku, Shinjuku-ku, Tokyo Japan

**Keywords:** Chimaera, Chimera, Lineage marker, Developmental neutrality, Reiterated transgene, *Tg(Hbb*-*b1)83Clo*

## Abstract

Recent reports of a new generation of ubiquitous transgenic chimaera markers prompted us to consider the criteria used to evaluate new chimaera markers and develop more objective assessment methods. To investigate this experimentally we used several series of fetal and adult chimaeras, carrying an older, multi-copy transgenic marker. We used two additional independent markers and objective, quantitative criteria for cell selection and cell mixing to investigate quantitative and spatial aspects of developmental neutrality. We also suggest how the quantitative analysis we used could be simplified for future use with other markers. As a result, we recommend a five-step procedure for investigators to evaluate new chimaera markers based partly on criteria proposed previously but with a greater emphasis on examining the developmental neutrality of prospective new markers. These five steps comprise (1) review of published information, (2) evaluation of marker detection, (3) genetic crosses to check for effects on viability and growth, (4) comparisons of chimaeras with and without the marker and (5) analysis of chimaeras with both cell populations labelled. Finally, we review a number of different chimaera markers and evaluate them using the extended set of criteria. These comparisons indicate that, although the new generation of ubiquitous fluorescent markers are the best of those currently available and fulfil most of the criteria required of a chimaera marker, further work is required to determine whether they are developmentally neutral.

**Electronic supplementary material:**

The online version of this article (doi:10.1007/s11248-015-9883-7) contains supplementary material, which is available to authorized users.

## Introduction

Experimental mouse chimaeras were first produced over 50 years ago (Tarkowski 1961) and remain useful for a wide range of studies (Eckardt et al. 2011). The utility of chimaeras depends on the availability of suitable markers for distinguishing the two cell populations that contribute to chimaeric tissues. Transgenic technology has provided both multi-copy transgenes, detectable by DNA in situ hybridisation (ISH), and a selection of transgenic reporter markers, which have been used with mouse chimaeras.

Early multi-copy transgenic markers included species-specific markers (Rossant et al. 1983), Y-chromosome-specific DNA markers (e.g. Patek et al. 1991) and an expressed multi-copy *cmyc* transgene that caused anatomical abnormalities and overgrowth in chimaeras (Augustin et al. 1998). However, the most widely used multi-copy DNA transgenic marker is the non-expressed, highly reiterated *Tg(Hbb*-*b1)83Clo* transgene (hereafter abbreviated to *Tg*), comprising approximately 1000 copies of the β-globin gene (Lo 1986; Lo et al. 1987; Katsumata and Lo 1988). This was first used in chimaeras over 25 years ago (Clarke et al. 1988; Thomson and Solter 1988), is available in ES cells (Ioffe et al. 1995; Quinn et al. 2010) and was widely used until quite recently (e.g. Quinn et al. 2010). Although this was once the preferred chimaera marker for many studies, it has now been superseded by reporter transgenes that are simpler to use and provide better spatial resolution.

The use of reporter transgenes as chimaera markers is not without problems, as expression may vary among tissues and some may show mosaic transgene expression. These problems led Swenson et al. to conclude that the older type of genotypic DNA markers, detectable in all nucleated cells types by DNA ISH, may be more suitable than reporter transgenes for cell transplantation studies (Swenson et al. 2007). However, improved markers have been produced more recently by introducing fluorescent protein transgenes into the *Rosa26* locus for use in mouse chimaera studies (Ueno and Weissman 2006; Ohtsuka et al. 2010, 2012). Qualitative analysis of chimaeras, with cells labelled with this type of marker, suggests that these newer reporter markers may overcome the shortcomings of previous ones (Ohtsuka et al. 2012). However, to our knowledge they have not been investigated quantitatively in chimaeras to determine whether they are developmentally neutral.

A cell lineage marker must be developmentally neutral, which means it must not change the properties of the marked cell, its progeny or its neighbours (Oster-Granite and Gearhart 1981; Kretzschmar and Watt 2012). Some reporter transgene markers affect growth and/or viability of non-chimaeric mice that are hemizygous or homozygous for the marker transgene (e.g. MacKay et al. 2005). Such effects may be mediated systemically but other effects may be cell autonomous. Thus, cells carrying a marker that is not quantitatively developmentally neutral might be at a selective disadvantage (or advantage) in chimaeras and those carrying a marker that is not spatially developmentally neutral might not mix normally with unmarked cells in the chimaera. Although quantitative and spatial aspects of developmental neutrality are critical criteria for chimaera markers, no consistent approach has been used to evaluate them and, in many cases, markers have been assumed to be developmentally neutral without sufficient quantitative experimental evidence.

It is important to develop a systematic approach for evaluating chimaera markers. A recent description of a new reporter marker for chimaeras included a qualitative comparison of spatial patterns (Ohtsuka et al. 2012). Characteristic tissue-specific spatial patterns previously reported for other chimaera markers were used as benchmarks for a qualitative assessment of spatial patterns produced in chimaeras using the new marker. In this case the new marker reproduced tissue-specific patterns reported for other markers but it is worth considering what could cause an abnormal pattern in order to understand what new information could be obtained using this comparative benchmarking approach. It seems unlikely that mosaic marker expression would alter the pattern qualitatively if mosaicism was established early in development and then stabilised, as this would simply be equivalent to producing chimaeras with a lower proportion of marked cells. However, the spatial pattern might be degraded if expression of the marker changed after the pattern was established (e.g. if expression of the marker was unstable). If there was no evidence of mosaic marker expression in non-chimaeric tissues, significant degradation of tissue-specific patterns could be explained by abnormal cell mixing. Benchmarking tissue-specific patterns could, therefore, provide a useful qualitative approach for investigating spatial aspects of developmental neutrality.

There is also a need to use quantitative methods to evaluate chimaera markers more objectively and, in particular, to test whether they are developmentally neutral. This prompted us to reconsider the criteria for chimaera markers by analysing several series of chimaeras that incorporated the older *Tg(Hbb*-*b1)83Clo* multi-copy marker. We are not advocating turning back the clock to use the multi-copy *Tg* marker in future chimaera studies but we hope that our analysis with this marker may help guide the evaluation of new markers. The multi-copy *Tg* marker has been used for many chimaera experiments but the only aspect of developmental neutrality that has been considered for this marker is the overall quantitative contribution of hemizygous *Tg/*− cells to the whole chimaeric fetus and various extraembryonic tissues (West et al. 1996). We, therefore, extended this approach to quantify the contribution of both hemizygous *Tg/*− and homozygous *Tg/Tg* cells to specific tissues in adult chimaeras.

The present study had three aims. The first aim was to develop a systematic approach that could be used for testing the new generation of chimaera markers for quantitative and spatial aspects of developmental neutrality. Work for this aim was divided into three sections. The first part involved an investigation of the viability of hemizygous *Tg/*− and homozygous *Tg/Tg* mice and the other two parts involved analysis of chimaeras. For the second part we did not use the multi-copy *Tg* marker itself as an endpoint but used independent markers that were also present in WT↔WT chimaeras. This allowed us to compare the quantitative contributions and spatial distributions of *Tg/Tg*, *Tg/*− and wild-type (WT) cells to adult and fetal *Tg/Tg*↔WT, *Tg/*−↔WT and WT↔WT chimaeras respectively.

For the final part of this first aim, we investigated the spatial distribution of *Tg*-positive cells using DNA ISH as the endpoint in three different tissues. We adopted the qualitative benchmarking approach used by Ohtsuka et al. (2012) and investigated whether the reiterated *Tg* marker could identify characteristic tissue-specific spatial patterns previously identified in mosaics and chimaeras using other markers. The multi-copy *Tg* marker is known to be a sub-optimal spatial marker because it produces only a small hybridisation signal and the transgene is not present in all sections of *Tg*-positive nuclei (Keighren and West 1993). This provided the opportunity to assess this qualitative benchmarking approach by investigating whether characteristic tissue-specific spatial patterns are significantly degraded using this sub-optimal marker. Previous studies of mosaics and chimaeras have shown that the two cell populations are distributed non-randomly in the adrenal cortex (Weinberg et al. 1985; Iannaccone 1987; Morley et al. 1996; MacKay et al. 2005), seminiferous tubules (Mizutani et al. 2005) and the neural retina (Reese et al. 1995, 1999). We, therefore, used the multi-copy *Tg* marker as an endpoint for spatial analysis in these three tissues in chimaeras. In addition we used chimaeras with cells that carry the *Tg* marker and were homozygous or heterozygous for the *Pde6b*^*rd1*^ retinal degeneration mutant allele to investigate the effects of losing most of the *Tg*-expressing cells after the spatial pattern has been established.

The second aim was to use the experience gained during these experiments to propose a systematic multi-step procedure for evaluating new chimaera markers against an extended set of marker criteria, comprising well-established ones (McLaren 1976; Oster-Granite and Gearhart 1981; West 1984; Rossant and Spence 1998) plus a more detailed assessment of developmental neutrality.

The third aim was to review published information about strengths and weaknesses of several different types of chimaera markers, using the same extended set of marker criteria, to determine whether the new *Rosa26* knock-in fluorescent chimaera markers fulfilled all the criteria or, at least, more than the other markers.

## Materials and methods

### Mice and production of chimaeras

All animal work was performed in accordance with institutional guidelines and UK Home Office regulations (licences PPL 60/1150 and PPL 60/1989). GLB mice, homozygous for three marker genes (*Tyr*^+*/*+^, *Gpi1*^*b/b*^, *Tg/Tg*), were produced on a mixed genetic background. “*Tg”* is used as the abbreviation for the multi-copy β-globin transgene *TgN(Hbb*-*b1)83Clo*) (Lo 1986; Lo et al. 1987; Katsumata and Lo 1988) and either “WT” (wild-type) or “−/−” is used to denote the absence of the transgene. BF1 mice were (C57BL/Ola × CBA/Ca)F1 hybrids (marker genotypes: *Tyr*^+*/*+^, *Gpi1*^*b/b*^, −*/*−), AAF1 mice were (BALB/c × A/J)F1 hybrids (marker genotypes: *Tyr*^*c/c*^, *Gpi1*^*a/a*^, −*/*−) and CF1 mice were (C57BL-*Gpi1*^*c*^*,Tyr*^*c*^/Ws × BALB/c-*Gpi1*^*c*^/Ws)F_1_ (marker genotypes: *Tyr*^*c/c*^, *Gpi1*^*c/c*^, −*/*−). *Tg/Tg, Tg/*− and −*/*− genotypes, produced by various crosses, were distinguished by DNA in situ hybridisation to β-globin in nucleated blood cells in blood smears (see below). A/J mice were purchased from HarlanOlac (Bicester, UK) and BALB/c mice were obtained from the Department of Medical Microbiology, University of Edinburgh. Other mice were bred and maintained, under conventional conditions in the Centre for Reproductive Biology, Edinburgh.

Chimaeras for analysis of developmental neutrality (fetal series CA and adult series AdCA) were made by aggregating 8-cell stage *Tyr*^+*/*+^, *Gpi1*^*b/b*^, (TGB × BF1)F2 embryos (which could be *Tg/Tg, Tg/*− or −*/*−) with 8-cell stage *Tyr*^*c/c*^, *Gpi1*^*a/a*^, −*/*− (BALB/c × A/J)F2 embryos as previously described (West and Flockhart 1994). Aggregated embryos were cultured overnight and E3.5 chimaeric embryos were transferred to the uteri of pseudopregnant CF1 (homozygous *Gpi1*^*c/c*^) females and the day of transfer was defined as E2.5 (according to when the recipient female mated with a vasectomised CF1 male). This produced pigmented, *Gpi1*^*b/b*^↔albino *Gpi1*^*a/a*^ chimaeras of three genotype combinations: *Tg/Tg*↔WT, *Tg/*−↔WT and WT↔WT. The genotype combination of each chimaera was identified retrospectively by DNA in situ hybridisation to the β-globin transgene.

Adult chimaeras used for spatial analysis of the retina (series AdCE) and for analysis of the effects of retinal degeneration (series AdCC) were produced in a similar way to those described above. The *Tg* marker was backcrossed onto the C3H/He strain, which carries the *Pde6b*^*rd1*^ retinal degeneration mutant allele (abbreviated to *rd1*), to produce *rd1/rd1, Tg/Tg, Tyr*^+*/*+^, *Gpi1*^*b/b*^ mice (stock name “RD”). Chimaeras in series AdCE were produced by aggregating (BF1 × RD)F1 embryos with (BALB/c × A/J)F2 embryos and were all *rd1/*+*, Tg/*−*, Tyr*^+*/*+^, *Gpi1*^*b/b*^ ↔ +/+, WT *Tyr*^*c/c*^, *Gpi1*^*a/a*^, where the heterozygous *rd1/*+ genotype has a WT (+/+) phenotype. Chimaeras in series AdCC were produced by aggregating (C3H × RD)F1 embryos with (BALB/c × A/J)F2 embryos and were all *rd1/rd1, Tg/*−*, Tyr*^+*/*+^, *Gpi1*^*b/b*^ ↔ +/+, WT *Tyr*^*c/c*^, *Gpi1*^*a/a*^.

Embryo aggregation was also used to produce ROSA26-*LacZ*^+*/*+^↔WT chimaeras, carrying the ubiquitously expressed ROSA26-*LacZ* transgenic reporter, TgR(ROSA26)26Sor (Friedrich and Soriano 1991) as described previously (Collinson et al. 2002).

### Analysis of fetal chimaeras

For analysis of fetal chimaeras (series CA), pregnant females were killed at E12.5 days gestation and the conceptuses were dissected into three epiblast-derived samples (fetus, amnion and visceral yolk sac mesoderm), two primitive endoderm derivatives (visceral yolk sac endoderm and parietal endoderm, attached to Reichert’s membrane) and two samples predominantly derived from the trophectoderm (placenta and a trophoblast sample dissected from Reichert’s membrane) as described elsewhere (West and Flockhart 1994; West et al. 1996). Physical parameters (numerical hind limb morphology index (McLaren and Buehr 1990; Palmer and Burgoyne 1991), crown-rump length and mass of the whole conceptus, fetus and placenta) were recorded as described previously (West et al. 1996). The proportion of pigmented cells in the RPE of each eye was estimated subjectively and averaged to provide an initial indication of the chimaeric composition.

Cell spreads or solid tissue samples were collected from the posterior of the fetus, including the hind limb, yolk sac and yolk sac endoderm and fixed in acetic alcohol (3 ethanol: 1 glacial acetic acid) for DNA in situ hybridisation to the β-globin transgene. These samples were used to identify chimaeric genotypes according to whether the conceptus contained cells with 2 hybridisation signals (*Tg/Tg*↔WT chimaeras), 1 hybridisation signal (*Tg/*−↔WT chimaeras) or no hybridisation signal (WT↔WT chimaeras), when pigment or GPI1 markers indicated a substantial contribution of (TGB × BF1)F2 cells, in fetal series CA. If preliminary evaluation of fetal eyes indicated a substantial contribution of pigmented cells, only the fetal samples were used to identify genotype combinations, otherwise yolk sac samples were also used. Fetal heads were fixed in acetic alcohol for eye histology and DNA in situ hybridisation to detect the β-globin transgene. Other samples were prepared for glucose phosphate isomerase (GPI) electrophoresis. The fetal trunk and placenta were stored at −20 °C, in 100 µl of distilled water or 50 % glycerol in water, in 1.5 ml microtubes. All other tissues from E12.5 chimaeras were stored in 10 µl of 50 % glycerol in microtest plates. Prior to electrophoresis, samples were lysed by three cycles of freeze/thawing with mechanical disruption.

### Analysis of adult chimaeras

Adult AdCA, AdCC and AdCE chimaeras were weighed at 1 and 3 months, the percentage coat pigmentation was estimated subjectively by two people and a blood sample was taken from the tail and used to prepare a blood smear for DNA in situ hybridisation and a sample for GPI electrophoresis. Adult AdCA chimaeras were classified as *Tg/*−*, Gpi1*^*b/b*^*, Tyr*^+/+^↔WT, *Gpi1*^*a/a*^*, Tyr*^c/c^ (overt coat colour chimaeras with one hybridisation signal in some nucleated blood cells), *Tg/Tg,Gpi1*^*b/b*^*, Tyr*^+/+^↔WT, *Gpi1*^*a/a*^*, Tyr*^c/c^ chimaeras (overt coat colour chimaeras with two hybridisation signals in some nucleated blood cells) or WT, *Gpi1*^*b/b*^*, Tyr*^+/+^↔WT, *Gpi1*^*a/a*^*, Tyr*^c/c^ chimaeras (overt coat colour chimaeras with significant GPI1B contributions in their blood samples but no hybridisation signal in nucleated blood cells). Most AdCC and AdCE chimaeras were killed at 3 months but AdCA were maintained until 6–7 months, when they were weighed again and killed. The percentage coat and eye pigmentation were estimated, a blood sample was collected (for preparation of blood smears and to provide a sample for GPI electrophoresis) and various tissues and organs were dissected, rinsed in PBS and blotted dry. Some were homogenised in distilled water with a Polytron homogeniser and stored at −20 °C in 1.5 ml microtubes for GPI electrophoresis and others, including eyes, adrenal glands, testes or dissected seminiferous tubules, were prepared for histology, DNA in situ hybridisation or β-galactosidase (β-gal) histochemistry as described below.

For GPI analysis, single samples were collected for the heart, lung, thymus, tongue, spleen, sub-maxillary plus parotid glands, pancreas, stomach, bone marrow and urinary bladder as well as a final blood sample. Both left and right samples were collected for kidneys, ovaries, oviduct, uterine horns, testes, epididymides, seminal vesicles, and posterior mammary fat pads. Multiple samples were collected for the brain (brain cerebrum and cerebellum plus medulla), liver (medial, left lateral, right lateral and caudal lobes), skeletal muscle (from all 4 limbs), small intestine (divided into three lengths) and the large intestine and caecum plus appendix. However, for reasons discussed in the “Results” section, we only included one of each pair or set of multiple samples in the final analysis. A final set of 18 samples from both sexes plus three female-specific samples or three male-specific samples was selected to evaluate the composition of the adult chimaeras by GPI electrophoresis. The 18 samples from both sexes comprised coat pigment (subjective estimate), left eye pigment (subjective estimate), brain (cerebrum), blood, spleen, left kidney, left hind limb muscle, tongue, heart, left mammary fat pad, stomach, small intestine (middle third), large intestine, liver (medial lobe), lung, pancreas, urinary bladder and sub-maxillary plus parotid glands. The three female-specific samples were left ovary, left oviduct, left uterine horn and the three male-specific samples were, left testis, left epididymis and left seminal vesicle. Thus, 21 of these 24 samples were used for GPI analysis for each sex.

### GPI electrophoresis

Cellulose acetate electrophoresis, staining for GPI activity and quantification of the % GPIB by scanning densitometry were carried out as previously described (West and Flockhart 1994). Maternal tissue (e.g. in placenta) produced only the GPI1C enzyme and was excluded from the analysis of the relative percentages of GPI1A and GPI1B allozyme bands (GPI1AA and GPI1BB homodimers). For chimaeric tissues, such as skeletal muscle and placenta, that produced a GPI1AB heteropolymer band, the percentage of GPI1 in the GPIAB heteropolymer band was divided equally between the GPI1A and GPI1B values and the final % GPI1B value was used for analysis. Images of the stained electrophoresis plates were obtained using a flatbed scanner (Epson V330 photo), cropped using Adobe Photoshop CS6 software and converted to high-contrast, greyscale images using the Auto Contrast function.

### Histology and DNA in situ hybridisation

Tissue samples for DNA in situ hybridisation (ISH) or eye histology for pigment analysis were fixed in acetic alcohol (3 ethanol: 1 acetic acid, v/v). After fixation, to facilitate sectioning, lenses were removed from the eyes, through a cut made in the cornea. The percentage of eye pigment was also estimated subjectively. Blood smears from AdCA chimaeras were fixed in acetic alcohol, air dried, immersed in acetone for 10 min, dehydrated through graded alcohols, air dried and used for DNA ISH to distinguish between *Tg/Tg*↔WT, *Tg/*−↔WT and WT↔WT chimaeras.

Most solid tissues were processed to paraffin wax for histology. Sections were cut at 7 µm thickness and mounted on glass microscope slides coated with 3-aminopropyltriethoxysilane (TESPA, Sigma) and de-waxed as described elsewhere (Keighren and West 1993). Fetal heads and adult eyes were stained with haematoxylin and eosin (H & E) for analysis of eye pigment by microscopy. Other tissue sections and blood smears were analysed by DNA ISH to the transgene and hybridised digoxygenin-labelled DNA probe was detected by diaminobenzidine (DAB) staining for peroxidase-labelled antibody as described previously (Keighren and West 1993).

Plastic Sections (3 µm) of adult chimaeric eyes were prepared for spatial analysis of pigmented patches (Hodson et al. 2011). The lengths of pigmented and albino patches in the retinal pigment epithelium (RPE) were measured in histological sections of fetal and adult chimaeric eyes and summarised as the “corrected mean patch length” and the median patch length for the minor cell population as described previously (Hodson et al. 2011). Patch lengths measured in µm were converted to estimates of cells per patch length using estimates of cell diameters for the E12.5 fetal RPE (estimated as 9.07 µm in the present study; data not shown) and adult RPE [estimated previously as 14.3 µm (West 1975)].

To visualise the contribution of *Tg*-positive cells to the adult adrenal cortex by ISH, a section close to the centre of the adrenal gland and a series of photographs was taken with a 25x lens (Leica Diaplan microscope) and a tiled montage of the whole adrenal cortex was assembled and all the ISH signals were marked on an overlay. Spatial distributions were evaluated subjectively but the proportion of *Tg*-positive cells was not estimated.

Histological sections of testes were used to analyse the distribution of *Tg*-positive cells in seminiferous tubules. Easily identifiable germ cells (mostly pachytene and large spermatocytes) were scored as *Tg*-positive or *Tg*-negative in 7 µm sections of seminiferous tubules, using a 10 × 10 eyepiece grid. The percentage of *Tg*-positive germ cell nuclei in chimaeras was corrected by dividing it by the proportion of *Tg*-positive germ cell nuclei in non-chimaeric, hemizygous *Tg*/− testes.

To analyse the contribution of *Tg*-positive cells to the adult retina by ISH, the mid-section and the two sections that were approximately halfway between the mid-section to the first or last section were selected for examination. Using a Leica Diaplan compound microscope and a 63x objective, a 10 × 10 eyepiece grid was positioned over the section so that the RPE crossed the 10 squares of a single row of the sampling grid and the neural retina was included in the rows above. The percentage of pigmented and albino RPE was estimated for each of these squares and averaged to produce a mean for the area covered by the 10 × 10 grid. The contribution of *Tg*-positive cells per unit area was calculated for the neural retina without counting *Tg*-negative cells. The number of *Tg*-positive nuclei in the outer nuclear layer (ONL), inner nuclear layer (INL) and ganglion cell layer (GCL) were counted for each of the 10 columns of the grid and used to calculate a total *Tg*-positive number for each cell layer for the whole field of view. Four widely separated 10 × 10 fields of view were analysed for each of the three sections to provide 12 fields of view per eye.

### X-gal staining of tissues from *LacZ*^+*/*+^↔WT chimaeras

For β-gal histochemistry of seminiferous tubules, the tunica albuginea was first removed and the testes were placed in 1 % (w/v) sodium citrate solution and the tubules gently unravelled (Meredith 1969). After 12 min they were rinsed in PBS and fixed in 0.2 % (w/v) glutaraldehyde solution at 4 °C for 1 h. Larger tissue samples were cut into small pieces before they were fixed in glutaraldehyde solution but adrenal glands were fixed whole. After fixation, samples were washed three times in “detergent wash” at room temperature and stained overnight at 37 °C in X-Gal staining solution as described previously (Collinson et al. 2002). After staining, tissues were washed in PBS, 3 % (v/v) dimethyl sulfoxide, post-fixed in 4 % (v/v) paraformaldehyde or acetic alcohol, washed again in PBS and either examined as whole mount samples or processed for wax histology (as above), sectioned at 7 µm, counterstained in H & E or neutral red and mounted under coverslips.

### Statistical analysis

Statistical tests indicated in the text and figure legends were performed using GraphPad Prism version 5.0c software. The choice of parametric or non-parametric tests was guided, in part, by D’Agostino-Pearson normality tests. Fisher’s exact tests and goodness of fit χ^2^ tests were performed using an on-line statistical calculator (http://vassarstats.net/). The error bars in the figures are 95 % confidence intervals (CI).

## Results

### Effect of the multi-copy transgene on viability of non-chimaeric *Tg/*− and *Tg/Tg* mice

Hemizygous *Tg/*− mice were produced at the expected Mendelian frequencies in reciprocal *Tg/*− × −*/*− crosses but there was a non-significant trend for fewer than expected *Tg/Tg* homozygotes in reciprocal *Tg/Tg* × *Tg/*− crosses and this was highly significant in *Tg/*− × *Tg/*− crosses (Online Resource 1; Supplementary Table S1). Overall the results indicate that survival to at least weaning age is normal for hemizygous *Tg/*− mice but reduced for *Tg/Tg* homozygotes.

### Production and physical comparisons of WT↔WT, *Tg/*−↔WT and *Tg/Tg*↔WT chimaeras

We used two additional chimaera markers, so we could evaluate developmental neutrality of the *Tg* marker by comparing how cells behave in chimaeras with and without the *Tg* marker. The GPI1 electrophoretic marker was used to investigate quantitative aspects of developmental neutrality and a pigment marker was used to compare cell mixing in control WT, *Gpi1*^*b/b*^, *Tyr*^+/+^↔WT, *Gpi1*^*a/a*^*, Tyr*^c/c^ chimaeras and two groups of experimental chimaeras: *Tg/*−*, Gpi1*^*b/b*^*, Tyr*^+/+^↔WT, *Gpi1*^*a/a*^*, Tyr*^c/c^ and *Tg/Tg,Gpi1*^*b/b*^*, Tyr*^+/+^↔WT *Gpi1*^*a/a*^*, Tyr*^c/c^ at both fetal and adult stages (Online Resource 2; Supplementary Figs. S1a, b).

**Fig. 1:**
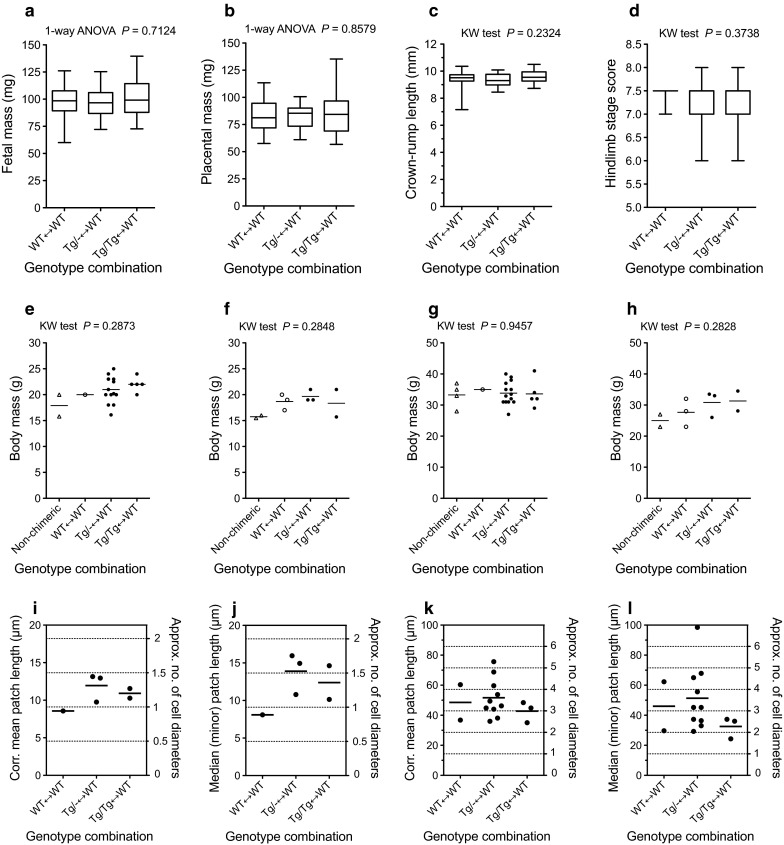
Comparison of physical parameters and spatial distributions of cells in WT↔WT, *Tg/*−↔WT and *Tg/Tg*↔WT chimaeras. **a**–**d** Comparisons of **a** fetal mass, **b** placental mass, **c** crown-rump length and **d** fetal maturity (hind limb development index) in WT (GPI1B)↔WT (GPI1A), *Tg/*− (GPI1B)↔WT (GPI1A), and *Tg/Tg* (GPI1B)↔WT (GPI1A) E12.5 fetal chimaeras [there were no significant differences among chimaeric genotypes by 1-way ANOVA for **a**, **b** or by Kruskal–Wallis (KW) tests for **c**, **d**. In the *box* and *whisker plots* the middle horizontal line is the median, the bottom and top of the *boxes* are first and third quartiles and the whiskers are minimum and maximum values]. **e**–**h** Comparisons of body mass in **e** males at 1 month, **f** females at 1 month, **g** males at 3 months and **h** females at 3 months for adult chimaeras and non-chimaeric siblings. There were no significant differences among groups by Kruskal–Wallis (KW) tests. **i**–**l** Estimates of the sizes of coherent clones of RPE cells in E12.5 fetal (**i**, **j**) and adult (**k**, **l**) chimaeras (shown as corrected mean patch length (**i**, **k**) or median patch length of the minor cell population (**j**, **l**). See text for explanation. Means are shown by *horizontal bars*. The mean RPE cell diameter is approximately 9.1 µm at E12.5 and approximately 14.3 µm in adults as shown by the *horizontal dotted lines*. 1-way ANOVAs showed no significant differences among chimaera genotypes in k (*P* = 0.5710) or l (*P* = 0.3792) and sample sizes in **i**, **j** were too small for meaningful statistical comparisons

Sixty-two conceptuses were identified as chimaeras at embryonic day (E) 12.5 (series CA): 20 WT ↔WT, 26 *Tg/*−↔WT and 16 *Tg/Tg*↔WT. There were no significant differences in physical parameters among the three groups of fetal chimaeras (Fig. 1a–d). Twenty-eight adult chimaeras (series AdCA) were produced: three female and one male WT, *Gpi1*^*b/b*^*, Tyr*^+/+^↔WT, *Gpi1*^*a/a*^*, Tyr*^c/c^, three female and 14 male *Tg/*−*, Gpi1*^*b/b*^*, Tyr*^+/+^↔WT, *Gpi1*^*a/a*^*, Tyr*^c/c^ and two female and five male *Tg/Tg,Gpi1*^*b/b*^*, Tyr*^+/+^↔WT, *Gpi1*^*a/a*^*, Tyr*^c/c^ chimaeras. Body mass did not differ significantly among four groups compared (three groups of chimaeras plus non-chimaeras) for either males or females at 1 or 3 months (Fig. 1e–h) although the low numbers of animals in some groups means this is not a powerful comparison.

### Quantitative comparisons of compositions of different groups of E12.5 chimaeric conceptuses

**Fig. 2:**
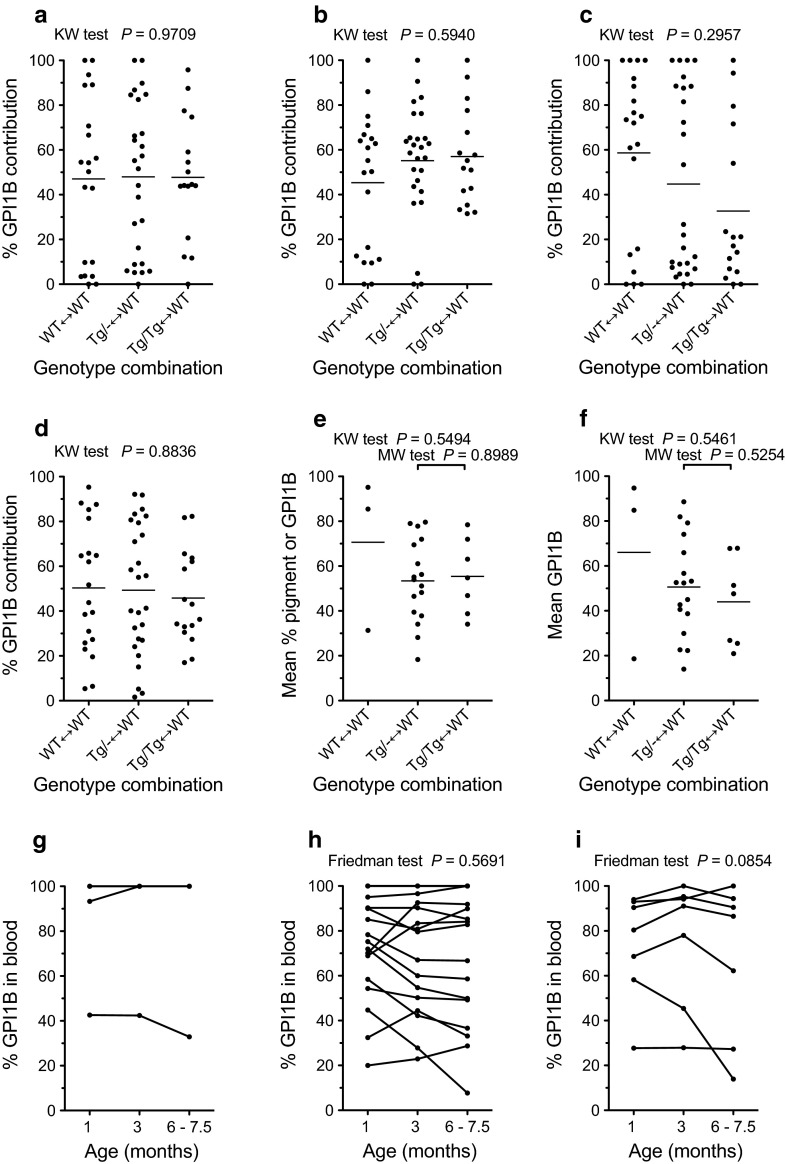
Comparison of composition of WT↔WT, *Tg/*−↔WT and *Tg/Tg*↔WT chimaeras. **a**–**d** Comparisons of composition of **a** fetus, **b** yolk sac endoderm, **c** placenta and **d** mean of all three samples (fetus, yolk sac endoderm and placenta), estimated as %GPI1B for E12.5 fetal chimaeras. There were no significant differences among chimaeric genotypes by Kruskal–Wallis tests. **e**, **f**. Comparisons of composition and distribution of individual values for representative tissues in different groups of adult chimaeras: **e** mean of 21 tissues (18 common to both sexes and 3 sex-specific tissues), **f** mean of 3 tissues (brain, left kidney, and liver). There were no significant differences among chimaeric genotypes by Kruskal–Wallis (KW) tests or by Mann–Whitney U-tests for the two larger groups. **g**–**i** Comparison of composition blood samples taken at different ages from the same adult chimaeras: **g** WT↔WT, **h** *Tg/*−↔WT and **i** *Tg/Tg*↔WT. There were no significant differences among ages in **h** or **i** by Friedman tests for repeated measures (and there were too few samples in g for a meaningful test)

To evaluate whether the *Tg* marker affects the composition of fetal chimaeras we compared the percentage of GPI1B (produced by *Gpi1*^*b/b*^ cells) in the fetus and extraembryonic tissues of the three groups of chimaeras using quantitative GPI electrophoresis. The original set of eight samples was simplified to three for the final analysis (Fig. 2a–d), as the composition of tissues within the same developmental lineage (epiblast, primitive endoderm or trophectoderm) were positively correlated for chimaeras of each of the three genotype combinations (Online Resources 2-4; Supplementary Figs. S1c & S2 and Supplementary Table S2). Distributions of the % GPI1B, analysed separately for the fetus, yolk sac endoderm and placenta, and for the mean of all three samples revealed no significant differences among the three chimaera genotypes (Fig. 2a–d) and all three groups included chimaeras with high and low GPI1B contributions. Thus, there was no evidence for cell selection against the hemizygous *Tg/*− or homozygous *Tg/Tg* genotype and by these criteria, both genotypes were considered to be quantitatively developmentally neutral overall for the E12.5 fetus and the extraembryonic tissues studied. For many purposes, it may not be necessary to separate the yolk sac endoderm and mesoderm so future quantitative comparisons of fetal chimaeras, with and without new markers, could be simplified to include just the fetus, whole yolk sac and placenta.

### Quantitative comparisons of compositions of different groups of adult chimaeras

We analysed the composition of adult chimaeras quantitatively using GPI electrophoresis (Online Resource 2; Supplementary Fig. S1a) to identify whether the presence of the multi-copy *Tg* marker caused cell selection or affected growth. As only four control WT↔WT chimaeras were recovered and one female died soon after 3 months, tissues were only available for GPI analysis from three control chimaeras for comparison with the other groups. Two of these WT↔WT chimaeras were predominantly pigmented and GPI1B and the other was predominantly albino and GPI1A. The deficiency of control WT↔WT chimaeras undermined the comparisons of the *Tg/*−↔WT and *Tg/Tg*↔WT groups with the controls but comparisons between the two experimental groups and quantitative analysis of the overall distributions within these groups still provided useful information. One objective of this part of the study was to identify a suitable combination of tissue samples to represent the composition of adult chimaeras. We analysed a large number of samples with the aim of identifying a smaller subset that could be used to simplify the evaluation of developmental neutrality.

We first considered whether separation of left and right sides of the body during gastrulation was likely to affect the composition of different samples from adult chimaeras. The compositions of most tissues in adult chimaeras are positively correlated with one another (Falconer et al. 1981) because much of the variability among chimaeras arises when the epiblast lineage separates from the primitive endoderm and trophectoderm (Falconer and Avery 1978; West et al. 1984). As the two genetically distinct cell populations in chimaeras are present very early in development and become finely intermixed before gastrulation occurs (Gardner and Cockroft 1998), the compositions of tissues on different sides of the body are unlikely to differ any more than samples from the same side. This is supported by two observations. First, although coat melanoblasts populate the skin from the neural crest independently on the left and right sides of the body and the antero-posterior distribution of coat pigmentation in pigmented↔albino chimaeras may vary between left and right sides, the overall percentage of pigment is usually similar in left and right sides (Online resource 5; Supplementary Fig. S3a–c). Second, analysis of the composition of four skeletal muscles samples, from left and right forelimbs and hindlimbs of 17 *Tg/*−*, Gpi1*^*b/b*^*, Tyr*^+/+^↔WT, *Gpi1*^*a/a*^*, Tyr*^c/c^ chimaeras, showed that correlations between samples from different sides of the body were no less significant than those from the same side (Online resource 5; Supplementary Fig. S3d–i). For these reasons we assumed that body side was not a confounding factor and that left and right samples were equivalent for purposes of analysis.

We next considered how to deal with paired tissue samples, such as kidneys, gonads or eyes. For chimaeras of some strain combinations, the composition of paired samples may be more closely related to one another than to other tissues, if they share tissue-specific selection pressures (Mintz and Palm 1969; Mintz 1970; West 1977). We did not investigate this in detail but, in case two paired samples differed less than two unpaired samples, we did not include both members of a pair as separate samples. Rather than use the mean value for paired samples, which might result in a lower variance for paired samples than unpaired samples, we only included one of each pair (left sample) in the analysis. Similarly, where we had multiple samples of other organs (e.g. liver lobes), we only included one in the final analysis to avoid confounding effects of greater similarities among samples from the same organs than from different organs (Vaux et al. 2012).

We chose not to use a 2-way analysis of variance (ANOVA) to look for differences among groups of chimaeras and tissues because there were so few WT↔WT chimaeras and the data were not normally distributed. To allow us to compare the overall composition of different groups of chimaeras, we calculated a mean contribution of GPI1B (or pigmented) cells for a panel of 21 tissues (see “Materials and Methods” section) for each chimaera. There were no significant differences among the three groups in the overall compositions of the chimaeras for these 21 tissues (Fig. 2e) by non-parametric Kruskal–Wallis tests. As there were only three control WT↔WT chimaeras, we also compared the overall composition of just the *Tg/*−↔WT and *Tg/Tg*↔WT chimaeras by Mann–Whitney U-tests and again there was no significant differences (Fig. 2e). Despite the limitations of the small size of the control WT↔WT group there was no evidence for a generalised cell selection against the hemizygous *Tg/*− or homozygous *Tg/Tg* genotype. This is consistent with the evidence for quantitative developmental neutrality from fetal chimaeras.

### Tissue-specific effects on tissue composition of adult chimaeras

**Fig. 3:**
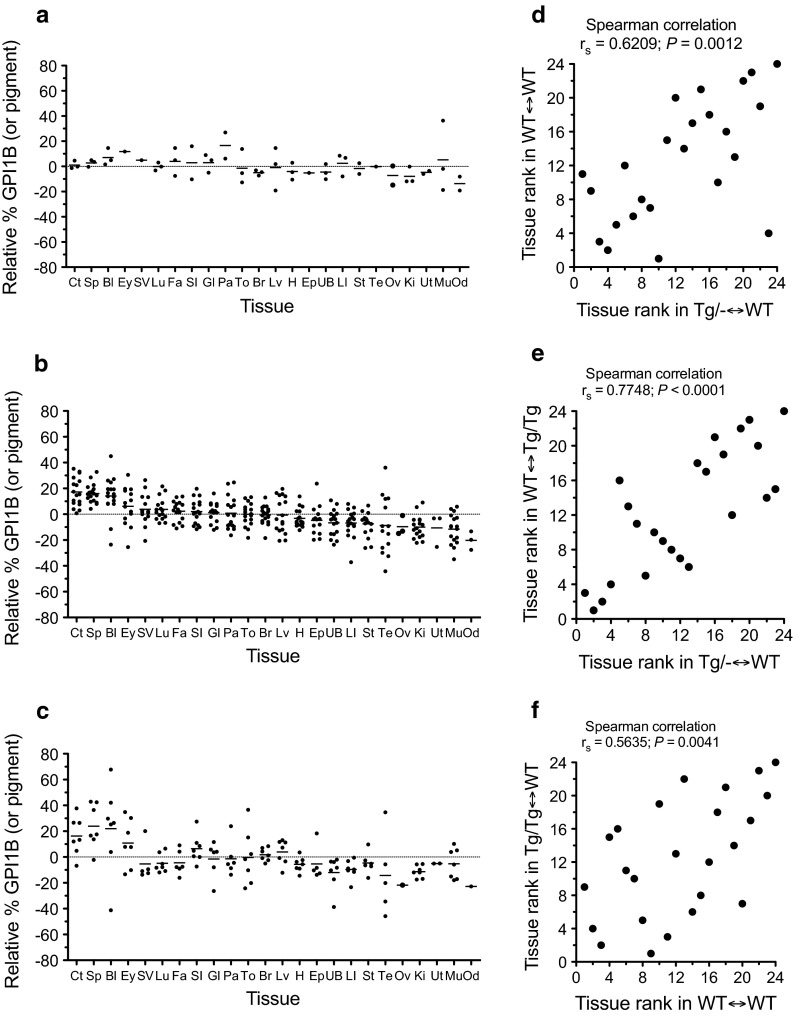
Comparisons of relative composition of different tissues in WT↔WT, *Tg/*−↔WT and *Tg/Tg*↔WT adult chimaeras. **a**–**c** The relative % GPI1B (or pigment) contribution to different tissues (calculated by subtracting the mean % GPI1B (or pigment) for all 24 tissues from the % GPI1B (or pigment) in the individual tissues separately for each chimaera) from **a** WT↔WT, **b** *Tg/*−↔WT and **c** *Tg/Tg*↔WT adult chimaeras. Tissues are ordered on the X-axis according to their relative % GPI1B values in *Tg/*−↔WT chimaeras. *Abbreviations* *Ct* coat pigment (subjective estimate), *Ey* eye pigment (subjective estimate), *Br* brain (cerebrum), *Bl* blood, *Sp* spleen, *Ki* left kidney, * Mu* left hind limb muscle, *To* tongue, *H* heart, *Fa* left mammary fat pad, *St* stomach, *SI* small intestine (middle third), *LI* large intestine, *Lv* liver (medial lobe), *Lu* lung, *Pa* pancreas, *UB* urinary bladder, *Gl* sub-maxillary and parotid glands, *Te* left testis, *Ep* left epididymis, *SV* left seminal vesicle, * Ov* left ovary, *Od* left oviduct, *Ut* left uterine horn. **d**–**f** Correlations of rank order of the 24 tissues according to their relative % GPI1B (or pigment) between **d** *Tg/*−↔WT and WT↔WT chimaeras, **e** *Tg/*−↔WT and Tg/Tg↔WT chimaeras and **f** WT↔WT and Tg/Tg↔WT chimaeras

To test whether the inclusion of the *Tg* marker had any tissue-specific effects on tissue composition we calculated the mean % GPI1B (or pigment) for each tissue for the three groups of chimaeras (Online Resource 2; Supplementary Fig. S1d). We compared the composition of pairs of tissues in a Spearman correlation matrix for the 17 *Tg/*−↔WT and seven *Tg/Tg*↔WT chimaeras (Online Resource 6; Supplementary Table S3) but not for the small group of three WT↔WT chimaeras. As expected from previous correlation analyses with chimaeras (Falconer et al. 1981) and reconstruction of cell lineage trees from somatic mutations (Wasserstrom et al. 2008; Salipante et al. 2010; Behjati et al. 2014), almost all the correlation coefficients were positive. Nearly all the correlations were statistically significant for the *Tg/*−↔WT chimaeras and many were significant for the *Tg/Tg*↔WT chimaeras. (It seems likely that the more significant correlations for *Tg/*−↔WT chimaeras reflects the larger group size rather than any effect of genotype combination.) Differences between tissues are illustrated in Fig. 3a–c, which shows the relative % GPI1B (or pigment) for 24 individual tissues (including both male-specific and female-specific samples) ranked according to their values for the large group of *Tg/*−↔WT chimaeras. This rank order showed a similar high to low trend in relative % GPI1B (or pigment) for WT↔WT and *Tg/Tg*↔WT chimaeras even though they have fewer chimaeras per group. Furthermore, the rank order of tissues was significantly positively correlated among all three groups as shown in Fig. 3d–f. (The most discrepant outliers occurred in the two correlations involving the small group of WT↔WT chimaeras; Fig. 3d, f). This implies that differences in composition among tissues are more likely to be attributable to genotype differences of the strain combination used to produce all three groups of chimaeras rather than the marker transgene.

The composition of the blood in *Tg/Tg* ↔WT chimaeras was not significantly correlated with any other tissues at 6–7.5 months (Online Resource 6; Supplementary Table S3b), raising the possibility of selection either for or against *Tg/Tg* blood stem cells over time. However, the composition of blood samples taken from chimaeras at different ages did not change significantly in a consistent direction between 1 month and 6–7.5 months in *Tg/*−↔WT or *Tg/Tg*↔WT chimaeras (Fig. 2g–i). There was, therefore, no evidence for consistent selection for or against *Tg/*− or *Tg/Tg* blood cells in chimaeras.

### Relationship between chimaeric composition and growth

The analysis of physical parameters (Fig. 1) revealed no obvious effects of *Tg* genotype on the size of fetal or adult chimaeras but the low numbers of adult WT↔WT chimaeras was a limitation. We, therefore, tested whether the size (mass) of individual chimaeras was associated with the overall contribution of *Tg/*− or *Tg/Tg* cells. Similar trends were seen in all groups of fetal chimaeras (including WT↔WT chimaeras), suggesting that any genetic effects on growth are more likely to be caused by genetic differences between the strains used to produce the chimaeras rather than the *Tg* marker transgene itself (Online Resource 7; Supplementary Fig. S4a–f). Fetal mass was weakly positively correlated with the fetal chimaeric composition in all three groups but this only reached significance for *Tg/Tg*↔WT chimaeras (*P* = 0.0412). Placental mass was significantly positively correlated with the placental composition for each of the chimaera combinations. Adult male and female chimaeras were considered separately and, as sample sizes were too small for meaningful analysis of females or WT↔WT males, the analysis was confined to *Tg/*−↔WT and *Tg/Tg*↔WT male chimaeras. As for the fetal chimaeras, body mass at 3 months was weakly associated with chimaeric composition (mean of 21 tissues; Online Resource 7; Supplementary Fig. S4g, h). This was non-significant for *Tg/*−↔WT chimaeras and, although it reached significance for *Tg/Tg*↔WT chimaeras (*P* = 0.0428), this was not significant without the smallest mouse (*P* = 0.1400). As all groups analysed showed similar trends, there is no convincing evidence for an affect on body size or growth that is mediated by the marker transgene rather than other genetic differences between the mouse strains used to produce the chimaeras.

### Simplified quantitative comparisons of different groups of adult chimaeras

As compositions of most adult tissues were positively correlated with one another in the largest group of chimaeras (Online Resource 6; Supplementary Table S3), a smaller subset of tissues should be adequate for future investigations of the overall quantitative developmental neutrality of chimaera markers. Coat pigmentation is a simple marker that is often used to assess the overall composition of adult chimaeras subjectively but it was not typical of the other 20 tissues analysed in the present study (Fig. 3b, c) and it is possible that pigmentation is often overestimated when it is assessed subjectively. Comparisons of estimates of chimaera composition using different combinations of tissue samples suggest that the mean % GPI1B in either a subset of 12 tissues or even just three tissues (brain, kidney and liver, representing predominantly ectoderm mesoderm and endoderm, respectively) instead of the full set of 21 tissues would be adequate (Online Resource 8; Supplementary Fig. S5). However, about 12 tissues would be more suitable for identification of tissue-specific effects and, if data were normally distributed, a 2-way ANOVA could be used to check for differences simultaneously among chimaera groups and among tissues (the tissues we chose for our subset of 12 excluded sex-specific tissues and subjective endpoints and comprised brain, blood, spleen, left kidney, left hind limb muscle, tongue, heart, small intestine, large intestine, liver, lung and pancreas). Furthermore, when the overall composition of each chimaera was calculated as the mean % GPI1B for either the subset of 12 tissues or the small subset of three tissues, there were still no significant differences among the different groups of chimaeras. Results for the full set of 21 tissues and the smallest subset of three tissues are shown in Fig. 2e, f. Similarly, for the intermediate subset of 12 tissues there were no significant differences among all three groups (*P* = 0.4177 by Kruskal–Wallis test) or between just the *Tg/*−↔WT and *Tg/Tg*↔WT chimaeras (*P* = 0.6566 by Mann–Whitney U-test).

### Effects of *Tg/*− and *Tg/Tg* genotypes on cell mixing in fetal and adult chimaeras

To evaluate whether the *Tg* marker affects the extent of cell mixing in chimaeric tissues we compared estimates of the sizes of coherent clones of pigmented and albino patches in the RPE in chimaeras with and without the *Tg* marker (Online Resource 2; Supplementary Fig. S1b). Coherent clone lengths were estimated in histological sections as both the “corrected mean patch length” (which corrects for effects of the proportion of pigmented cells on the patch length) and the uncorrected median patch length for the minor cell population. Both estimates have been shown to produce similar results although the median values may be larger if the minor population is close to 50 % (Hodson et al. 2011). In all three groups of E12.5 fetal chimaeras, the mean coherent clone sizes in the RPE were equivalent to approximately 0.95–1.3 cell diameters (0.89–1.7 cell areas), implying that the cells were finely intermixed but in adults the corrected mean patch length had increased to about 3.0–3.6 cell diameters (9.0–13.0 cell areas) (Fig. 1i–l). These results are consistent with a previous report of mean coherent clone sizes in the RPE of approximately 1.3 cell areas at E12.5 and 5.7–10.5 in adults for other control chimaeras without a transgenic lineage marker (West 1976). Median patch lengths for the minor cell population (Fig. 1j, l) were generally comparable to the corrected mean patch lengths (Fig. 1i, k). Too few E12.5 chimaeras were analysed for a meaningful statistical analysis but the results showed that cell mixing was extensive in all three groups. For adults neither the corrected mean patch lengths nor the median patch lengths for the minor cell population differed significantly among the three groups by 1-way ANOVA. Thus, this analysis showed no evidence that the presence of *Tg/*− or *Tg/Tg* cells in the chimaeras significantly affected the extent of cell mixing in the RPE.

### Identification of spatial patterns in the adrenal cortex of adult chimaeras with the *Tg* marker

The use of independent markers allowed us to evaluate both quantitative and spatial aspects of developmental neutrality (described above) but this does not indicate whether the multi-copy *Tg* marker itself provides good quantitative and spatial information. We next tested whether the *Tg* marker could be used to identify previously characterised patterns in chimaeric tissues. We compared the distributions of clonal lineages that occur as radial stripes in the adrenal cortex and segments in the seminiferous tubule in adult *Tg/*−↔WT with those identified in previous studies with other markers and compared them directly to patterns produced by the β-gal reporter transgene in *LacZ*↔WT chimaeras.

**Fig. 4:**
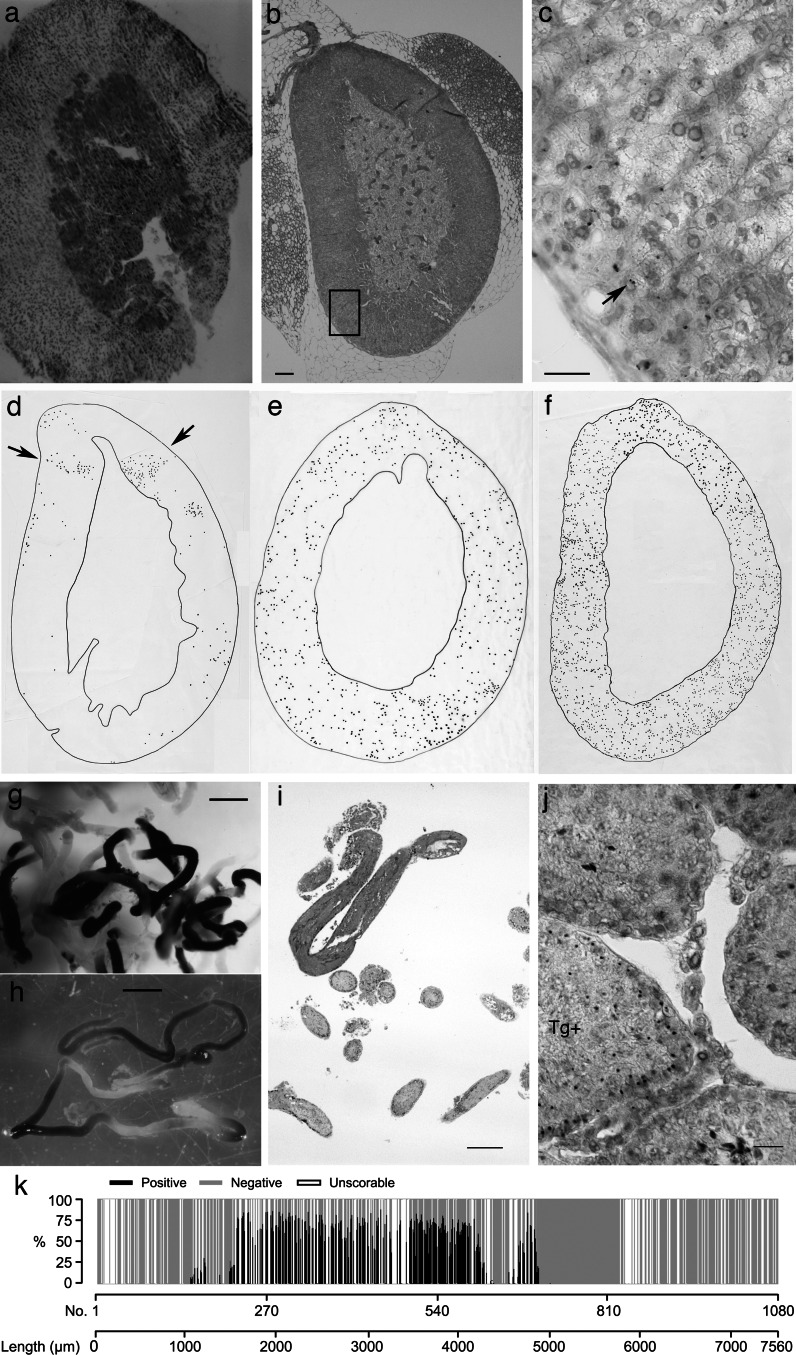
Comparison of spatial patterns in *LacZ*↔WT chimaeras and *Tg*↔WT chimaeras. **a** Radial pattern of β-gal-positive (*blue*) and negative stripes in the adrenal cortex of a *LacZ*↔WT chimaera (the adrenal medulla appears entirely β-gal-positive but this could be a technical artefact; see text). **b**, **c** A section of an adrenal gland from *Tg/Tg*↔WT chimaera AdCA6, following DNA ISH and light H&E staining. The region boxed in **b** is shown at a higher magnification in **c** in order to visualise nuclei with *brown* in ISH signals. Some nuclei have two ISH signals (*arrow*) as expected for *Tg/Tg* homozygous cells. The field of view is too small to identify whether radial stripes are present when high magnification is used to visualised ISH signals. **d**–**f** Tracings of the distributions of ISH signals in *Tg*-positive nuclei in adrenal cortices from tiled photographic images of sections of adrenal glands from three *Tg*↔WT chimaeras with different proportions of *Tg*-positive cells, following DNA ISH. (Adrenals: d, *Tg/*−↔WT chimaera AdCC26; e, *Tg/Tg*↔WT chimaera AdCC20; f, *Tg/Tg*↔WT chimaera AdCA6). Stripes of *Tg*-positive cells are only obvious in the adrenal cortex with the lowest proportion of Tg-positive cells (**d**). **g**, **h** β-gal-positive (*blue*) and negative lengths in seminiferous tubules dissected from testes of *LacZ*↔WT chimaeras. **i** Histological section of seminiferous tubules dissected from a *LacZ*↔WT chimaera, stained for β-gal and then embedded in paraffin wax. **j** A section of testis from *Tg/Tg*↔WT chimaera AdCA33, following DNA ISH and light H&E staining. *brown* ISH signals are present in the section of tubule labelled Tg + but not in the other tubules. **k** Analysis of the percentage of *Tg*-positive germ cells in a 7560 µm length of seminiferous tubule, comprising 1080 tubule sections in 443 serial testis sections from *Tg/*−↔WT chimaera AdCA33. *Scale bars* 20 µm (**c**, **j**) 100 µm (**b**), 200 µm (**i**), 1 mm (**g**, **h**). (Color figure online)

The adrenal cortex of *LacZ*↔WT chimaeras showed a pattern of radial stripes (Fig. 4a) as previously demonstrated for various chimaeras and mosaics (Weinberg et al. 1985; Iannaccone 1987; Morley et al. 1996; MacKay et al. 2005). In *Tg/Tg*↔WT and *Tg/*−↔WT adrenals, *Tg*-positive nuclei were not visible at the low magnification required to view the whole cortex so it was difficult to see the pattern unless it was traced from a montage of tiled photographs (Fig. 4b–f). However, stripes of *Tg*-positive cells were only obvious in the adrenal cortex with the lowest proportion of *Tg*-positive cells (Fig. 4d). The poor resolution of the expected spatial pattern is probably mainly because the *Tg* marker is not detected in all cells [some nuclear sections from *Tg*-positive nuclei have no ISH signal because, when histological sectioning bisects a nucleus, the target DNA may be confined to one nuclear section (Keighren and West 1993)].

Rather surprisingly, the medulla of the *LacZ*↔WT chimaeric adrenal shown in Fig. 4a appeared to be entirely β-gal positive and was typical of other *LacZ*↔WT chimaeras analysed by this method. However, the medullas of *Tg*↔WT chimaeras contained both *Tg*-positive and *Tg*-negative cell populations (data not shown) suggesting the uniform β-gal staining in the medulla was an artefact. β-gal staining was not done on frozen sections but on intact adrenal glands, which had been lightly fixed in gluteraldehyde. These were then post-fixed after staining and processed to paraffin wax for histology. The adrenal medulla expresses endogenous β-galactosidase (Diliberto et al. 1976) so uniform β-gal staining in the medulla of chimaeras could represent endogenous β-gal activity (particularly if gluteraldehyde failed to penetrate to the medulla) and/or diffusion of the reporter β-gal stain during tissue processing through solvents and hot wax. Nevertheless, it is notable that the radial striped pattern in the adrenal cortex, which has been identified using both β-gal staining on frozen sections (Morley et al. 1996) and other markers (Weinberg et al. 1985; MacKay et al. 2005), was also clearly detected in wax sections of β-gal stained adrenals (Fig. 4a).

### Identification of spatial patterns in seminiferous tubules of adult chimaeras with the *Tg* marker

β-gal staining of whole seminiferous tubules dissected from testes of *LacZ*↔WT chimaeras revealed a 1-dimensional pattern of alternating lengths of β-gal positive and β-gal negative regions (Fig. 4g, h), similar to that described for GFP↔WT chimaeras (Mizutani et al. 2005). These observations imply that the marked and unmarked germ cell populations were not finely intermingled but formed large coherent clones within the tubules. Consistent with this, the germ cells were usually entirely β-gal positive or all β-gal negative in most histological sections of the stained tubules (Fig. 4i), notwithstanding the possibility of stain diffusion during processing, as discussed above for the adrenal medulla.

The *Tg* marker is unsuitable for analysis of whole mount tissues, so one seminiferous tubule was analysed by DNA ISH in serial sections and the spatial distribution of *Tg*-positive germ cells was reconstructed. After preliminary scan of sections from one testis from each of ten chimaeras, the right testis of *Tg/Tg*↔WT chimaera AdCA33 was chosen for analysis as it contained both *Tg*-positive and *Tg*-negative germ cells and the left testis had 40.2 % GPI1B (*Tg*-positive cell population). DNA ISH was performed on serial 7 µm sections and one tubule was followed through almost all of 443 sections (apart from a few that were unscoreable for technical reasons). The tubule looped back on itself twice so the 443 testis sections contained 1080 sections of this particular tubule (equivalent to 7560 µm). Most of the germ cells scored were pachytene or large spermatocytes as they are readily identifiable as germ cells. These were scored as *Tg*-positive or *Tg*-negative and, in most sections they were either all *Tg*-negative or predominantly *Tg*-positive (Fig. 4j). This is illustrated by the bar chart of the corrected percentage *Tg*-positive germ cells in each section of the tubule, which represents the reconstructed length of seminiferous epithelium This shows that most of the *Tg*-positive germ cells are grouped into three patches, 175, 2940 and 336 µm in length (Fig. 4k). The grouping of most *Tg*-positive germ cells into large patches shows that the *Tg* marker is capable of revealing the expected pattern seen on whole mounts even though the method is too laborious to be useful for routine spatial analysis.

### Identification of the spatial relationships between different regions of the retina in adult chimaeras with the *Tg* marker

We next examined the neural retina. The inner nuclear layer (INL), outer nuclear layer (ONL) and ganglion cell layer (GCL) of the retina are formed by lamination of a single cell layer in the optic cup and previous studies with other markers in chimaeras and mosaics have shown that clones of cells span the thickness of the neural retina although some cell types are also dispersed laterally (Reese et al. 1995, 1999). The retinal pigment epithelium (RPE) overlies the neural retina but adjacent cells of the RPE and neural retina are not closely related because these two layers arise from separate regions of the optic vesicle and only adopt their final positions after the prospective neural retina invaginates to form the optic cup. We, therefore, predicted that the distribution of *Tg*-positive cells in the ONL, INL and GCL of chimaeras should be spatially related with one another but not with those in the RPE. Previous studies have shown that the *Tg* marker can identify radial stripes of marked cells across the width of the neural retina in fetal chimaeras, before delamination of the different layers is complete (Collinson et al. 2001), but it was not known whether this marker could be used to identify the adult pattern after lateral dispersion has occurred.

**Fig. 5:**
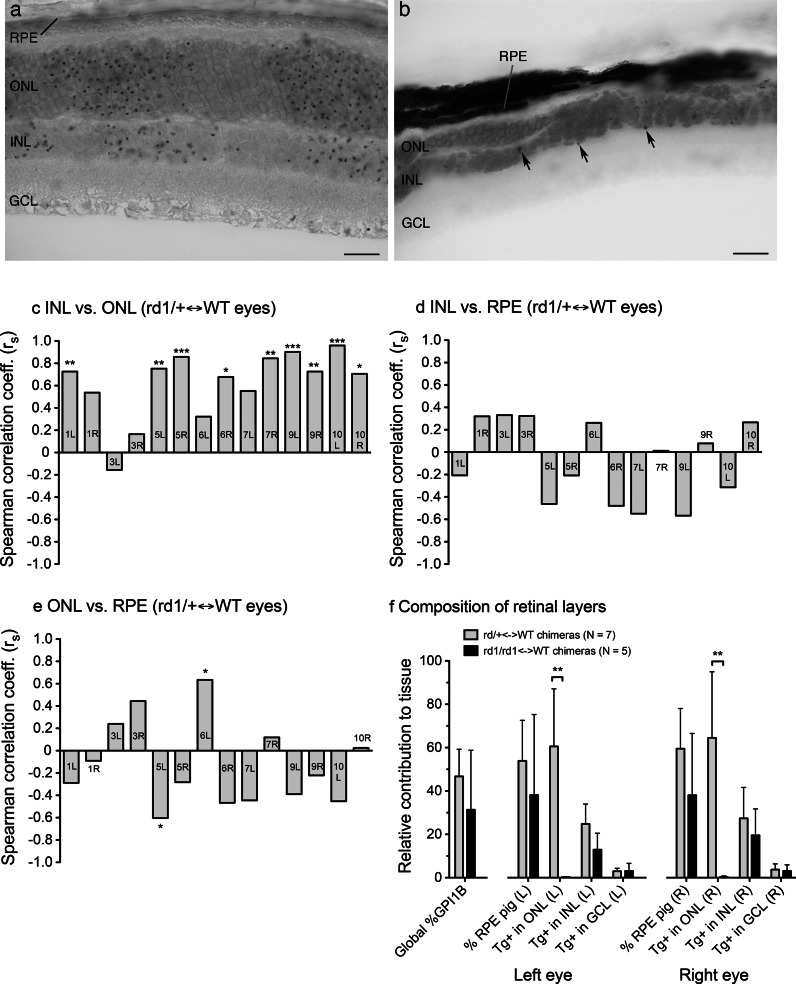
Distribution of *Tg*-positive cells in histological sections of retinas of adult *Tg/*−↔WT chimaeras with and without retinal degeneration. **a**, **b** In situ hybridisation (ISH) to multi-copy *Tg*-marker on histological sections. **a** Distribution of hybridisation signals (*brown spots* in nucleus) shows *Tg*-positive cells are arranged in broad stripes across the width of an adult *Tg/*−*, Tyr* ^+*/*+^↔WT, *Tyr* ^*c/c*^ retina without retinal degeneration. **b** An adult *rd1/rd1, Tg/*−*, Tyr* ^+*/*+^↔+/+, WT, *Tyr* ^*c/c*^ chimaeric retina after degeneration of *rd1/rd1* cells in the ONL. The ONL is thin and uneven and *Tg*-positive cells are present in the INL; some *brown* hybridisation signals are indicated with *arrows* (the section is not flat so the GCL and some hybridisation signals are out of focus). **c**–**e** Spearman correlation coefficients (r_s_) for pairwise comparisons of the contributions of *Tg*-positive cells to three retinal tissues (mean number of *Tg*-positive cells per field of view in the INL and ONL and the % pigment in the RPE) for 12 fields of view for each of 14 eyes analysed from 7 *rd1/*+↔WT chimaeras (series AdCE) without retinal degeneration. The heights of the bars show the r_s_ correlation coefficients, the statistical significance is shown above or below each bar (*P < 0.05; **P < 0.01; ***P < 0.001) and symbols within each bar refer to the eye analysed; e.g. “1L” is chimaera AdCE1, left eye. **f** Quantitative comparisons of the relative composition of different tissues in adult control *rd1/*+↔WT (series AdCE without retinal degeneration) and experimental *rd1/rd1* ↔WT chimaeras (series AdCC with retinal degeneration), showing mean estimated global % GPI1B (mean of individual values for brain, kidney and liver) and, separately for left and right eyes the, mean % pigmented RPE and mean % *Tg*-positive cells per field of view in the ONL, INL and GCL. The *bar chart* shows the mean ± 95 % confidence intervals for 11–12 fields of view for each eye (12 fields of view were analysed for each eye except the right eye of *rd1/rd1*↔WT chimaera AdCC19). The number of *Tg*-positive cells per field of view was significantly greater in the *rd1/*+↔WT chimaeras (without retinal degeneration) than the *rd1/rd1*↔WT chimaeras (with retinal degeneration) for the ONL of both left and right eyes by Mann–Whitney U-test (**P < 0.01 in each case) but not for any other tissues (*Abbreviations* *GCL* ganglion cell layer, *INL* inner nuclear layer, *L* left, *ONL* outer nuclear layer, *pig* pigmented, *R* right, *RPE* retinal pigment epithelium). *Bar* in **a**, **b** = 20 µm. (Color figure online)

We analysed seven adult *rd1*/+, *Tg/*−*, Tyr*^+*/*+^↔+/+, WT, *Tyr*^*c/c*^ chimaeras, from series AdCE, to test whether pigmented (*Tyr*^+*/*+^) cells in the RPE and *Tg*-positive cells in different layers of the neural retina were distributed as predicted from their developmental origins. (Although the *Tg/*−*, Tyr*^+*/*+^ cells were also heterozygous *rd1/*+ for the *rd1* retinal degeneration allele, this had no phenotypic effect, as *rd1* is recessive.) As expected, *Tg*-positive regions of the outer nuclear layer (ONL) and inner nuclear layer (INL) usually appeared to be quite well radially aligned with one another but showed no obvious spatial relationship with the pigmented regions of the RPE (Fig. 5a). This was borne out by quantitative comparisons showing that the mean numbers of *Tg*-positive nuclei per field of view in the ONL and INL were almost always positively correlated (13/14 eyes) and this was usually statistically significant (9/13 eyes) (Fig. 5c). In contrast, positive and negative correlations of ONL or INL with the % pigmented RPE occurred with similar frequencies and were rarely significant (Fig. 5d, e). Thus, the predicted spatial relationships were detectable, even though the *Tg* marke*r* is sub-optimal for spatial analysis.

### Survival of some *rd1/rd1* cells in the outer nuclear layer after retinal degeneration

The distribution of *Tg*-labelled retinal cells was also used to investigate the effects of losing most of the *Tg*-positive ONL cells (photoreceptors) after the spatial pattern has been established. To do this we produced a group of five adult *rd1/rd1, Tg/*−*, Tyr*^+*/*+^↔+/+, WT, *Tyr*^*c/c*^ chimaeras (series AdCC), in which the *Tg*-positive cells were all homozygous for the *Pde6b*^*rd1*^ retinal degeneration mutation (abbreviated to *rd1*). DNA ISH was then used to determine whether any *rd1/rd1, Tg/*− photoreceptors survived after retinal degeneration. Retinal degeneration occurs by 3 weeks in homozygous *rd1/rd1* mice, involving death of rod cells followed by a more gradual degeneration of cone cells (Carter-Dawson et al. 1978; Han et al. 2013). Previous chimaera studies showed that the ONL (photoreceptor layer) varies in thickness in retinas of adult *rd1/rd1*↔+/+ chimaeras (Mintz and Sanyal 1970; Wegmann et al. 1971; LaVail and Mullen 1976; West 1976). It was originally assumed that all the *rd1/rd1* photoreceptors in the ONL would die, so the surviving ONL cells would all be WT. However, *rd1/rd1* photoreceptor survival can be prolonged by treatment with survival factors (Han et al. 2013) and it is possible that, in a chimaeric retina, the neighbouring WT cells might rescue some of the *rd1/rd1* photoreceptors. We, therefore, investigated if any *rd1/rd1*cells in the ONL survived to 3 months and, if so, whether the original pattern of broad stripes across the neural retina could still be detected.

The ONL was thinner than normal in most *rd1/rd1*↔+/+, AdCC chimaeras (Fig. 5b). Quantitative comparisons showed that, the contribution of *Tg*-positive cells to the ONL was greatly reduced in *rd1/rd1*↔+/+ chimaeras compared to the *rd1/*+↔+/+ chimaeras (Fig. 5f). For the left eyes of 7 *rd1/*+↔+/+ chimaeras, the mean number (±95 %CI) of *Tg*-positive cells in the ONL per field of view was 60.6 ± 21.3 whereas for the left eyes of 5 *rd1/rd1*↔+/+ chimaeras it was only 0.27 ± 0.17 (Mann–Whitney U-test; *P* = 0.0057). For right eyes the equivalent contributions were 64.5 ± 24.4 and 0.34 ± 0.34 (*P* = 0.0057). The composition of the other retinal layers did not differ significantly between the two groups. In case global differences in composition between the two groups of chimaeras affected the results shown in Fig. 5f, the analyses were repeated after dividing the mean RPE, ONL, INL and GCL (ganglion cell layer) results for each chimaeric eye by the mean % GPI1B. The ONL remained the only tissue that showed a significant difference between the two groups of chimaeras (data not shown). Thus, as expected, the *Tg/*−, *rd1/rd1* cells were severely depleted in the ONL but not in other retinal layers.

The depletion of *Tg/*−, *rd1/rd1* cells in the ONL was so extensive that it altered the tissue morphology. The consequences were too severe to provide a useful model for investigating the effects of depleting the number of marked cells on an established spatial pattern. However, the survival of a small proportion of *Tg*-positive *rd1/rd1* cells in the ONL of these chimaeras raises an interesting question about their identity. As noted earlier, rod photoreceptors are expected to degenerate by 3 weeks, unless they can be rescued by neighbouring WT cells, but cone cells degenerate more gradually. Further studies with both lineage and cell type-specific markers will be required to identify whether the small number of surviving homozygous *rd1/rd1* cells in the ONL of *rd1/rd1*↔+/+ chimaeras are *rd1/rd1* rod cells that have been rescued by the presence of WT retinal cells or *rd1/rd1* cone cells that have survived until 3 months.

## Discussion

### Evaluation of developmental neutrality of the *Tg* marker

We found no evidence that the *Tg* marker had a significant effect on growth of fetal or adult chimaeras but viability of non-chimaeric, homozygous *Tg/Tg* mice was reduced. As the transgene is not expressed, this suggests either that its production created an insertional mutation or that the very high copy number has a deleterious genomic effect, which affects viability of homozygotes. For example, this transgene is known to form heterochromatin in at least some cell types (Manuelidis 1991) and this could affect the expression of neighbouring genes.

A large number of samples were analysed with an independent quantitative marker (GPI electrophoresis) for both fetal and adult chimaeras to investigate whether hemizygous *Tg/*− and homozygous *Tg/Tg* cells are at a selective advantage or disadvantage or whether they are quantitatively developmentally neutral. Quantitative comparisons of the tissue composition of *Tg/*↔WT, *Tg/Tg*↔WT and WT↔WT chimaeras and longitudinal comparisons of the composition of blood sampled at different ages provided no evidence that *Tg/*− or *Tg/Tg* cells have a selective advantage or disadvantage compared to WT cells. We have also suggested how this part of the analysis could be simplified using fewer tissues but sufficient should be included to avoid undermining investigations of tissue-specific selection.

We also used RPE pigmentation as an independent spatial marker to investigate cell mixing quantitatively. This showed that the pigmented patch sizes were comparable in the RPE of *Tg/*↔WT, *Tg/Tg*↔WT and WT↔WT chimaeras, implying that the marker did not significantly affect cell mixing in this tissue. Although a larger study would be required to ensure adequate statistical power, this proof of principle study showed that this approach could be used to investigate spatial developmental neutrality.

### Qualitative comparative analysis of tissue-specific patterns

The *Tg* marker itself was used to determine whether characteristic tissue-specific spatial patterns reported for other markers could be identified in *Tg*↔WT chimaeras. The predicted tissue-specific patterns were identified in the seminiferous tubules and neural retina of *Tg*↔WT chimaeras, albeit with more difficulty than with some other markers. For the adrenal cortex, however, the expected pattern of radial stripes was only identified in samples with a low proportion of *Tg*-positive cells. Although the small size of the ISH signal may contribute to the difficulty in detecting the expected spatial pattern, the main problem is likely to be that the target DNA is not present in all sections of *Tg*-positive nuclei. If so, this suggests that qualitative comparisons of tissue-specific patterns might be capable of identifying pattern degradation caused by an unstable marker, which continues to be lost in some cells after the pattern is established. However, we have not tested whether this approach can identify altered cell mixing. Although the *Tg* marker has already been used to detect major defects in cell mixing in chimaeras caused by reduced or absent Pax6 (e.g. Quinn et al. 1996; Collinson et al. 2001), it remains unclear whether a purely qualitative method would be effective for detecting more subtle changes in cell mixing.

### A five-step procedure for evaluating new chimaera markers

As noted earlier, there is a need to develop a rigorous, systematic approach for evaluating new transgenic markers used for chimaeras. Our investigations with the multi-copy *Tg* marker in fetal and adult chimaeras illustrates how developmental neutrality can be investigated objectively and we suggest these types of investigations should be included when assessing new transgenic markers for use in chimaera studies. Consequently, we propose the following five-step procedure for evaluating new chimaera markers against the set of 14 criteria shown in Table 1. Most of these criteria have been discussed by others (McLaren 1976; Oster-Granite and Gearhart 1981; Rossant and Spence 1998) but we consider developmental neutrality in more detail.

**Table 1:** Evaluation of different types of chimaera markers using an expanded set of marker criteria

Criteria	Marker					
	Pigment	GPI1 allozymes	Multi-copy *Tg(Hbb*-*b1)83Clo*	*Rosa26*-*LacZ* *Gt(ROSA)26Sor*	Fluorescent reporters	
					Addition transgene	*Rosa26* knock-in^a^
A. Cellular location characteristics of marker						
1. Cell localised (not secreted extracellularly)	+	−^b^	+	+	+	+
2. Cell autonomous (not transferred between cells or affecting other cells)	+	+	+	+	+	+
B. Marker detection						
3. Detection is simple and efficient	+	+	–	+	+	+
4. Detectable in histological sections of fixed specimens	+	−	+	+	+	+
5. Detectable, non-invasively in live adults	+	−	−	−	+^c^	+^c^
6. Detectable in live preimplantation embryos	−	−	−	−	+	+
7. Provides quantitative information	+	+	±^d^	+	+ or ±^e^	+
8. Provides spatial resolution at the single cell level	+	–	±^d^	+ or ±^f^	+ or ±^e^	+
C. Cell and tissue distribution of marker						
9. Present in all tissues (ubiquitous at the tissue level)	−	+	+	+^g^	+ or −^e^	+
10. Present in all cells (ubiquitous at the cell level; no mosaic expression)	+	+	±^d^	+	+ or −^e^	+
D. Effects on viability, development and growth						
11 No effect on viability	+	+	+ or −^h^	+ or −^i^	+ or −^j^	+ or −^k^
12 No effect on development and growth	+	+	+^l^	+^m^	+ or −^n^	+ or −^k^
E. Cellular effects on developmental neutrality						
13 Quantitatively, cell-lineage neutral: does not cause cell selection	(+)	(+)	+^o^	NK	+^p^ or NK	NK
14 Spatially, cell-lineage neutral: does not affect cell mixing	(+)	(+)	+^q^	NK	NK	NK

+, yes; (+), assumed to be yes; ±, yes but sub-optimal; −, no; NK, not known; GFP, green fluorescent protein; GPI1, Glucose phosphate isomerise-1; ISH, in situ hybridisation, WT, wild-type

^a^See Ohtsuka et al. (2012) and Ryu et al. (2013)

^b^GPI1 enzyme is cell localised but spatial information is lost during analysis by electrophoresis

^c^Fluorescent markers are detectable in skin

^d^ISH signal not detectable in every nuclear section so quantification requires positive controls and single-cell resolution is imperfect

^e^Few addition transgenes show genuine ubiquitous expression and some show mosaic expression so compromising quantification and single-cell resolution

^f^Diffusion, poor stain penetration or endogenous activity may compromise single cell resolution

^g^May not be expressed in olfactory bulb granule cells (Zambrowicz et al. 1997)

^h^Homozygous *Tg/Tg* mice are not all viable (present study)

^i^Homozygous *Rosa26* ^*LacZ/LacZ*^ mice are not all viable (Zambrowicz et al. 1997)

^j^Few reports and results vary for different fluorescent markers: DsRed1 is toxic (Hadjantonakis et al. 2002) and homozygous *TgTP6.3tauGFP* ^+*/*+^ mice with tauGFP fusion protein are not viable (MacKay et al. 2005) but others may be viable

^k^Homozygous *Rosa26* ^*CAG*−*tdTomato*^ mice are retarded at birth and most die within 4 weeks (Ohtsuka et al. 2012)

^l^No significant physical differences between chimaeras with and without the *Tg* marker and the weak correlation between body mass and chimaeric composition is not attributable to the *Tg* marker (present study and West et al. 1996)

^m^Heterozygous *Rosa26* ^*LacZ/*+^ mice show no overt phenotype (Zambrowicz et al. 1997)

^n^Hemizygous *TgTP6.3tauGFP* ^+*/*−^ mice with tauGFP fusion protein are small (MacKay et al. 2005) but others may be normal

^o^ *Tg/*− and *Tg/Tg* cells showed no selective disadvantage (present study and West et al. 1996)

^p^Hemizygous *TgTP6.3tauGFP* ^+*/*−^ cells showed no selective disadvantage in fetal chimaeras (MacKay et al. 2005)

^q^ *Tg/*− and *Tg/Tg* cells showed no effects on cell mixing (present study)

*Step 1: Review of published information*. The first step would be to review what is known about the type of marker to determine whether it is likely to fulfil criteria 1–8 in Table 1. For example, preliminary assessment of a new knock-in GFP marker that is designed to be ubiquitous could rely on what has been established about cell localisation (criterion 1) and cell autonomy (criterion 2) from other GFP transgenic mice, unless the new marker is a novel GFP fusion protein. Most transgenic markers will fulfil criteria 1 and 2, but double-labelled chimaera experiments will be required to test them rigorously (see step 5) and re-evaluate the preliminary conclusions of step 1. Step 1, also involves reviewing if it is known whether the marker is easily detected and able to provide all the types if information required for the intended experiment, even if it does not fulfil all criteria 4-8. This can be supplemented by other investigations described in step 2.

*Step 2: Evaluation of marker detection.* Non-chimaeric animals that carry the marker should be tested, both to answer any questions about criteria 3-8 that were not answered in step 1 and to ensure that the marker is present in all tissues (criterion 9) and in all cells of each tissue (criterion 10). Ubiquitous expression at the cellular level is particularly important as mosaic expression is common among transgenic animals produced by random transgene integration (Dobie et al. 1997) and this can undermine analysis of chimaeric tissues. It is well known that genetic background can alter transgene expression so this step should be done using mice with the same genetic background that will be used to produce the chimaeras. Additional experiments with double-labelled chimaeras are also recommended (see step 5).

*Step 3: Genetic crosses to check for effects on viability and growth.* This step involves crosses to produce non-chimaeric mice that are homozygous and heterozygous for the marker plus WT siblings to enable comparisons of viability (criterion 11) and growth (criterion 12). A simple means of analysing viability is shown in Online Resource 1; Supplementary Table S1. To investigate growth, it is recommended that body mass (and perhaps other physical phenotypes) should be compared either at a specific age or at intervals (e.g. MacKay et al. 2005).

*Step 4: Comparisons of chimaeras with and without the marker.* Although the new marker (NM) could itself be used for assessing quantitative and spatial behaviour, this would preclude any comparisons between WT↔NM and WT↔WT chimaeras. As we consider this is necessary to evaluate developmental neutrality rigorously, we recommend that step 4 should involve producing chimaeras, all of which have one WT cell population. In some chimaeras the second cell population will carry the new marker plus an independent quantitative marker (QM) and an independent spatial marker (SM): WT↔[NM, QM, SM] chimaeras. In other chimaeras the second cell population will just carry the independent markers: WT↔[QM, SM] chimaeras. (Alternatively, of course, the same independent marker could be used as the quantitative and spatial marker.) Ideally, the developmental neutrality of marker transgenes should be determined separately for XY↔XY, XX↔XY and XX↔XX chimaeras but, if the sex chromosome composition is not investigated, it may be sufficient to compare male and female chimaeras separately for most non-reproductive tissues. However, this is not the perfect solution because, although most XX↔XY chimaeras will develop as adult males, some may develop as females or hermaphrodites, depending on the proportion of XY cells in the somatic tissues of the gonad (Mullen and Whitten 1971; McLaren 1984; Bradbury 1987).

Growth and physical parameters should be compared for the different groups of chimaeras with and without the new marker to further evaluate criterion 12 (growth) as shown in Fig. 1a–h. The independent quantitative marker should be used to compare the composition of a range of tissues in chimaeras with and without the marker being tested, as shown for *Gpi1* polymorphisms in Fig. 2a–f. This provides a means of investigating whether the new marker causes cell selection or is quantitatively developmental neutral (criterion 13). If data are normally distributed, differences among chimaera groups and tissues could be analysed by a 2-way ANOVA. Cell selection may also be identified by comparing the composition of blood sampled at different ages. To help assess effects of the marker on development and growth (criterion 12), growth and physical parameters should also be related to the overall composition of individual chimaeras, as described in Online Resource 7; Supplementary Fig. S4. The independent spatial marker should be used to analyse spatial distributions using a rigorous quantitative method (e.g. Fig. 1i–l). This provides a means of investigating whether the marker affects cell mixing or is spatially developmental neutral (criterion 14). To further evaluate the spatial aspects of developmental neutrality, it may also be useful to select some well-characterised, tissue-specific spatial patterns, established as benchmarks with other chimaera markers, and compare these qualitatively to the equivalent patterns produced with the new marker, as described by Ohtsuka et al. (2012).

*Step 5: Analysis of chimaeras with both cell populations labelled.* In this step chimaeras should be produced where the new marker labels one cell population and an independent spatial marker labels the second cell population: NM↔SM chimaeras (Kwiatkowski et al. 2009; Ohtsuka et al. 2012; Ryu et al. 2013). If any cells are unlabelled this will imply that one or both markers show mosaic expression, so fail to fulfil criterion 10 (ubiquitous expression at the cellular level). This would provide a more rigorous test than the simple analysis proposed in step 2. Conversely, any double-labelled cells would imply that one or both of the markers is transferred between cells, either in vivo or during tissue processing. Unless this is confined to tissues where cell fusion occurs naturally, one or both markers would fail criterion 2. By the end of step 5 it should also be possible to re-evaluate the evidence from step 1 and decide whether the marker can provide the required quantitative and spatial information (criteria 7 and 8).

### Review of chimaera markers

Table 1 compares the characteristics of the multi-copy *Tg* marker with several types of reporter transgene chimaera markers using the expanded set of 14 criteria, discussed above. GPI1 and pigment markers are also included as we used them in the present study.

Differences between *Tyr*^+^ and *Tyr*^*c*^ pigment markers can be detected easily in the coat, to identify overt chimaeras, and in histological sections of pigmented tissues, such as the retinal pigment epithelium (RPE), where it is cell-localised and provides an excellent spatial marker (Sanyal and Zeilmaker 1977). However, use of pigment as a marker is restricted to the small number of pigmented tissues. GPI1 electrophoresis is straightforward (Nagy et al. 2008) and can be used quantitatively with the common *Gpi1*^*a*^ and *Gpi1*^*b*^ alleles that were used in the present study, provided care is taken to avoid overstaining which may overestimate the minor band. Detection by enzyme electrophoresis involves tissue disruption so cell localisation is lost during analysis.

The *Tyr*^*c*^ albino mutation and different *Gpi1* polymorphisms segregate in genetic crosses according to the expected Mendelian ratios and there is no evidence of impaired viability, fertility, development or growth in heterozygotes or homozygotes. Cells with different *Tyr* or *Gpi1* genotypes can make both high and low contributions to chimaeras and the overall balance in a series of chimaeras depends on the strain combination (Mullen and Whitten 1971; Falconer and Avery 1978; West and Flockhart 1994). Furthermore, there is no evidence of cell selection in chimaeras made from congenic strains carrying different *Gpi1* alleles (Behringer et al. 1984). Comparison of the composition of pigmented and unpigmented tissues also provides no evidence that pigment markers cause cell selection in pigmented tissues (West et al. 1997). Furthermore, pigmented and unpigmented cells mix extensively in the fetal RPE without any evidence of cell sorting (West 1976). Overall, it is very unlikely that pigment or the two common GPI1 variants causes cell selection or affects the extent of cell mixing but this has not been investigated rigorously.

Detection of the multi-copy *Tg(Hbb*-*b1)83Clo* transgene by DNA ISH requires fixed tissues and is labour-intensive. As already noted, it is sub-optimal for spatial analysis on histological sections because, although it is present in all nucleated cells the reiterated target DNA is not present in all nuclear sections (Keighren and West 1993). Nevertheless, quantitative analysis is possible and is improved by use of tissue-specific correction factors (based on analysis of positive control sections) and/or normalisation with control tissues from the same chimaera (Jagerbauer et al. 1992; Quinn et al. 1996; Crosby et al. 1998; Collinson et al. 2000; Manuel et al. 2007). The present study revealed no evidence that the *Tg/*− or *Tg/Tg* genotype affected body size or the composition of the fetus, placenta, extraembryonic membranes or adult tissues in chimaeras or caused selection of blood cells. This implies that the multi-copy *Tg* transgenic marker is quantitatively developmentally neutral in both hemizygotes and homozygotes. However, the evidence for reduced viability of non-chimaeric, homozygous *Tg/Tg* mice indicates that it would be prudent to only use hemizygous *Tg/*− cells in chimaeras, until the effects of the homozygous *Tg/Tg* genotype are better understood.

The *Gt(ROSA)26Sor* gene trap transgene (commonly known as ROSA26-*LacZ* or *Rosa26*^*LacZ*^) (Friedrich and Soriano 1991; Zambrowicz et al. 1997) produces *E. coli* β-galactosidase (β-gal) and has been widely used as a chimaera marker. This does not show mosaic transgene expression and β-gal expression is essentially ubiquitous at both tissue and cellular levels, although one report indicates it is not expressed in olfactory bulb granule cells (Zambrowicz et al. 1997). The β-gal enzyme is relatively easy to detect by X-gal histochemistry but not by immunostaining (Brazelton and Blau 2005). The high temperatures required for wax embedding means X-gal histochemistry cannot be used on wax sections but frozen sections or small pieces of intact tissues can be stained then post-fixed and wax-embedded for sectioning. However, substrate penetration of intact tissue samples is limited, diffusion may compromise single-cell resolution even if samples are lightly fixed before staining, and care has to be taken to avoid confusion with endogenous mouse β-gal activity (Brazelton and Blau 2005; Bolon 2008). Nevertheless, the methods are sufficiently robust to fulfil criteria 1–4, and 7–10 in Table 1 (with a few caveats). Neither heterozygous *Rosa26*^*LacZ/*+^ nor homozygous *Rosa26*^*LacZ/LacZ*^ mice has an overt phenotype but while heterozygotes were produced at the expected Mendelian frequencies, fewer than expected homozygotes were recovered (Zambrowicz et al. 1997). We are not aware of any reports of systematic investigations of the cellular aspects of developmental neutrality for this marker.

The first generation of fluorescent transgenic markers for chimaeras were randomly integrated addition transgenics with ubiquitous promoters driving expression of a fluorescent reporter, such as EGFP (enhanced green fluorescent protein) (Okabe et al. 1997; Ikawa et al. 1998; Hadjantonakis et al. 1998). Some fluorescent reporters were expressed as fusion proteins and/or targeted to different sub-cellular regions but an exhaustive review is beyond the scope of this Discussion. A significant limitation of this type of fluorescent marker is that expression often varies among tissues and some tissues may show mosaic expression (MacKay et al. 2005; Ohtsuka et al. 2012). Furthermore, the expression level or the frequency of transgene-expressing cells may decline with time or after changing the genetic background (Brazelton and Blau 2005). The problem of mosaicism, associated with the expression of randomly integrated fluorescent marker transgenes, has been overcome recently by production of fluorescent transgenes targeted to the endogenous *Rosa26* locus and strains with widespread and uniform expression have now been described which fulfil most of the criteria in Table 1 (Ohtsuka et al. 2010, 2012).

The unique advantage of fluorescent markers is that they are detectable in living cells by direct fluorescence microscopy, so are particularly suitable for preimplantation stage chimaeras (Hadjantonakis et al. 1998; MacKay and West 2005; Ohtsuka et al. 2012). Fluorescent imaging of chimaeric tissue sections also provides good spatial information (MacKay et al. 2005; Eberhard and Jockusch 2010; Ohtsuka et al. 2012; Ryu et al. 2013). It is also possible to label both cell populations in a chimaera with different coloured fluorescent markers (Kwiatkowski et al. 2009; Ohtsuka et al. 2012; Ryu et al. 2013) or produce chimeras with more than two labelled cell populations, using multiple fluorescent markers (Ueno and Weissman 2006; Ohtsuka et al. 2012). Many naturally fluorescent markers can also be detected by immunofluorescence, which may improve the sensitivity, or immunohistochemistry, which can be combined with standard histology for better resolution of the non-fluorescent tissue architecture and a more permanent endpoint that permits archiving slides for future data mining. A ubiquitous fluorescent marker that is detectable by direct fluorescence and immunofluorescence or immunohistochemistry is, therefore, the most versatile of those currently available.

There is some information about effects of fluorescent marker transgenes on viability. For example, it has been suggested that high intracellular GFP levels may compromise normal physiology (Brazelton and Blau 2005). Also, the DsRed1 marker is toxic (Hadjantonakis et al. 2002) but variants DsRed.T3 (Vintersten et al. 2004) and DsRed2 (Ohtsuka et al. 2012; Ryu et al. 2013) are not. Most mice homozygous for *Rosa26* knock-in of *CAG*-*tdTomato* die by 4 weeks (Ohtsuka et al. 2012), *TgTP6.3tauGFP*^+*/*+^ homozygotes, expressing tauGFP fusion protein, are not viable and *TgTP6.3tauGFP*^+*/*−^ hemizygotes are small (MacKay et al. 2005). Nevertheless, contributions of *TgTP6.3tauGFP*^+*/*−^ cells to fetal *TgTP6.3tauGFP*^+*/*−^↔WT chimaeras, using balanced and unbalanced strain combinations, suggested that the hemizygous cells were quantitatively developmentally neutral (MacKay et al. 2005). Where distributions of cells, expressing various fluorescent chimaera markers, have been compared to patterns produced with other chimaera markers, they appear similar (MacKay et al. 2005; Ohtsuka et al. 2012). However, the extent of cell selection and cell mixing in chimaeras, marked with fluorescent markers, have rarely been studied quantitatively or compared to other chimaeras, using independent markers so there is little information about developmental neutrality.

## Conclusions

We analysed chimaeras carrying the multi-copy *Tg* marker to develop objective criteria for testing whether a marker is developmentally neutral. We have suggested how this approach could be simplified and incorporated into a 5-step procedure to evaluate other chimaera markers using 14 criteria. Our review of chimaera markers implies that the fluorescent transgenes driven by the endogenous *Rosa26* locus (Ohtsuka et al. 2012) fulfil more of our criteria than the other markers that we considered and if they prove to be developmentally neutral they would fulfil all our marker criteria.

## Electronic supplementary material

Supplementary material (PDF 63 kb)

Online Resource 1 (PDF 68 kb)

Online Resource 2–5 (PDF 4717 kb)

Online Resource 6 (PDF 138 kb)

Online Resource 7 & 8 (PDF 196 kb)
