# Supplementary material for: Lessons from mouse chimaera experiments with a reiterated transgene marker: revised marker criteria and a review of chimaera markers
Source: Transgenic Res. 2015 Jun 6;24(4):665–91. doi: 10.1007/s11248-015-9883-7 (PMC4504987; doi:10.1007/s11248-015-9883-7)
Supplement: Supplementary file 1 — Supplementary material (PDF 63 kb) [file 11248_2015_9883_MOESM1_ESM.pdf]

ELECTRONIC SUPPLEMENTARY MATERIAL

Online Resource 1 (Supplementary Table S1). Production of -/- (WT), *Tg/-* and *Tg/Tg* mice from different crosses

Online Resource 2 (Supplementary Fig. S1). Composition of individual tissues of WT↔WT, *Tg/-*↔WT and *Tg/Tg*↔WT chimaeras.

Online Resource 3 (Supplementary Fig. S2). Relationships among individual tissues within each of the three primary developmental lineages in individual E12.5 fetal chimaeras.

Online Resource 4 (Supplementary Table S2). Correlation matrices showing Spearman correlation coefficients (r_s) for % GPI1B in fetus and extraembryonic tissues of E12.5 fetal chimaeras

Online Resource 5 (Supplementary Fig. S3). Relationships in composition of samples from left and right side of bodies of adult chimaeras.

Online Resource 6 (Supplementary Table S3). Correlation matrices showing Spearman correlation coefficients (r_s) for % GPI1B (or % pigment) in representative tissues in adult *Tg/-* ↔WT and *Tg/Tg* ↔WT chimaeras

Online Resource 7 (Supplementary Fig. S4). Relationships between mass and composition of WT↔WT, *Tg/-*↔WT and *Tg/Tg*↔WT chimaeras

Online Resource 8 (Supplementary Fig. S5). Comparisons of compositions of different combinations of tissue samples from WT↔WT, *Tg/-*↔WT and *Tg/Tg*↔WT adult chimaeras
