# Supplementary material for: Lessons from mouse chimaera experiments with a reiterated transgene marker: revised marker criteria and a review of chimaera markers
Source: Transgenic Res. 2015 Jun 6;24(4):665–91. doi: 10.1007/s11248-015-9883-7 (PMC4504987; doi:10.1007/s11248-015-9883-7)
Supplement: Supplementary file 2 — Online Resource 1 (PDF 68 kb) [file 11248_2015_9883_MOESM2_ESM.pdf]

**Online Resource 1 (Supplementary Table S1) Production of -/- (WT), *Tg/-* and *Tg/Tg* mice from different crosses**

| Cross (female × male) | Observed Offspring Number | | | Observed Offspring Percentage | | | Expected Percentage | | | *P*-values ($\chi^2$ test) | | | |
|---|---|---|---|---|---|---|---|---|---|---|---|---|---|
| | -/- | *Tg/-* | *Tg/Tg* | -/- | *Tg/-* | *Tg/Tg* | -/- | *Tg/-* | *Tg/Tg* | *Tg/-* vs. -/- | *TgTg* vs. *Tg/-* | *TgTg* vs. -/- | Overall |
| 1a -/- × *Tg/-* | 40 | 51 | 0 | 44.0 | 56.0 | 0 | 50 | 50 | 0 | $P = 0.294$ (NS) | NA | NA | NA |
| 1b *Tg/-* × -/- | 155 | 152 | 0 | 50.5 | 49.5 | 0 | 50 | 50 | 0 | $P = 0.888$ (NS) | NA | NA | NA |
| Total 1a+b | 195 | 203 | 0 | 49.0 | 51.0 | 0 | 50 | 50 | 0 | $P = 0.729$ (NS) | NA | NA | NA |
| 2a *Tg/-* × *Tg/Tg* | 0 | 35 | 21 | 0 | 62.5 | 37.5 | 0 | 50 | 50 | NA | $P = 0.082$ (NS) | NA | NA |
| 2b *Tg/Tg* × *Tg/-* | 0 | 31 | 26 | 0 | 54.4 | 45.6 | 0 | 50 | 50 | NA | $P = 0.597$ (NS) | NA | NA |
| Total 2a+b | 0 | 66 | 47 | 0 | 58.4 | 41.6 | 0 | 50 | 50 | NA | $P = 0.091$ (NS) | NA | NA |
| 3 *Tg/-* × *Tg/-* | 169 | 292 | 87 | 30.8 | 53.3 | 15.9 | 25 | 50 | 25 | $P = 0.144$ (NS) | $P < 0.0001$ | $P < 0.0001$ | $P < 0.0001$ |

Frequencies of genotypes produced by reciprocal crosses do not differ significantly by Fisher's Exact test for crosses 1a and 1b ($P = 0.2850$) or crosses 2a and 2b ($P = 0.4468$). NA, not applicable.
