# Supplementary material for: Lessons from mouse chimaera experiments with a reiterated transgene marker: revised marker criteria and a review of chimaera markers
Source: Transgenic Res. 2015 Jun 6;24(4):665–91. doi: 10.1007/s11248-015-9883-7 (PMC4504987; doi:10.1007/s11248-015-9883-7)
Supplement: Supplementary file 3 — Online Resource 2–5 (PDF 4717 kb) [file 11248_2015_9883_MOESM3_ESM.pdf]

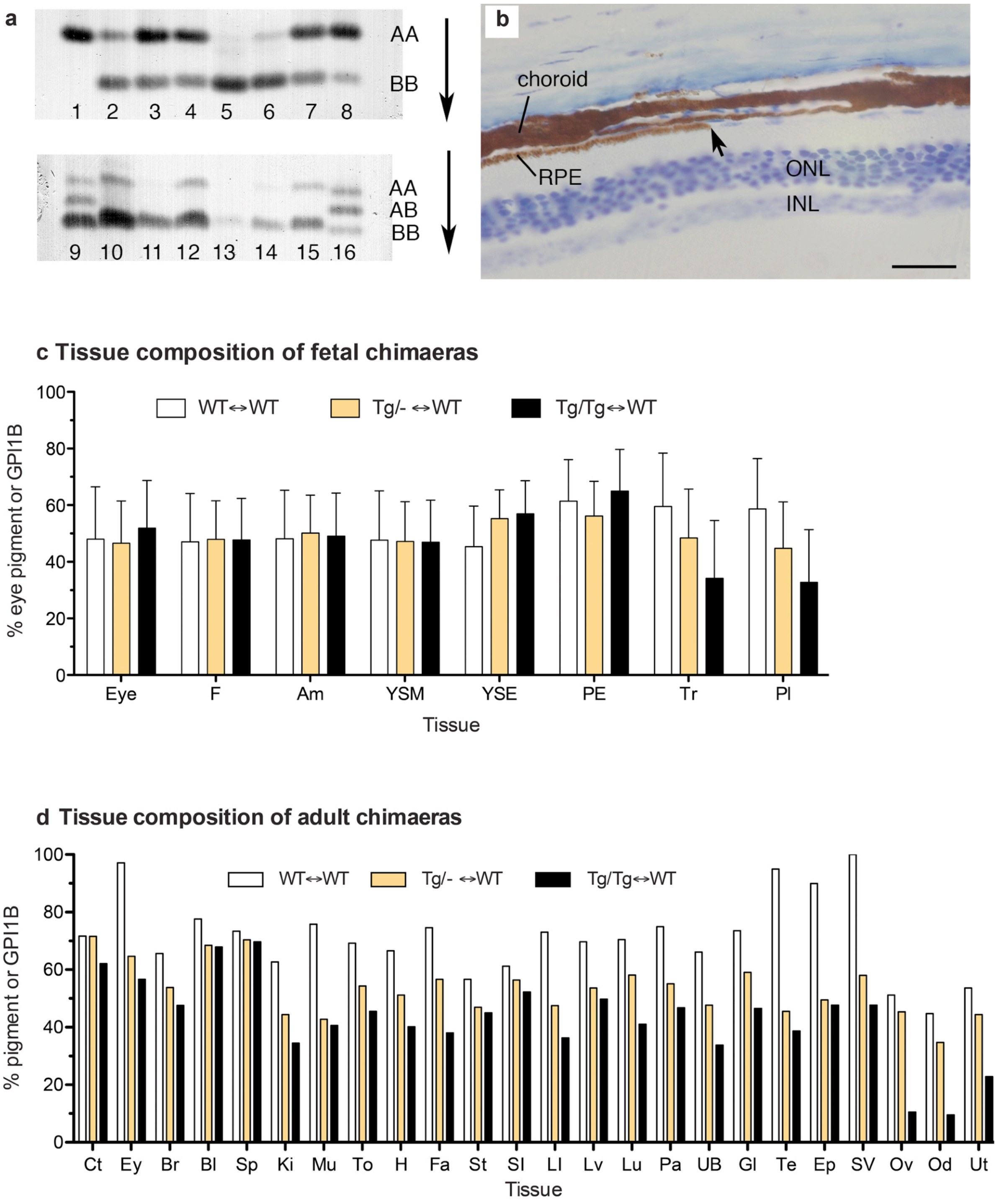

Online Resource 2 (Supplementary Fig. S1). Composition of individual tissues of WT↔WT, *Tg/-*↔ WT and *Tg/Tg*↔WT chimaeras.

Online Resource 2 (Supplementary Fig. S1). Composition of individual tissues of WT↔WT, *Tg/-*↔ WT and *Tg/Tg*↔WT chimaeras.

a. Two GPI electrophoresis plates with chimaera samples. Top plate shows results for 3-month blood samples from eight putative AdCA adult chimaeras. Lane 1, WT, *Gpi1*$^{a/a}$ non-chimaera. Lanes 2- 8, different AdCA *Gpi1*$^{b/b}$ ↔*Gpi1*$^{a/a}$ chimaeras, which were classified as WT↔WT (lane 3), *Tg/-*↔ WT (lanes 2, 4, 6, 7 & 8) and *Tg/Tg*↔ WT (lane 5) respectively. Lower plate (lanes 9-15) shows results for several tissues from WT*, Gpi1*$^{b/b}$↔WT*, Gpi1*$^{a/a}$ chimaera AdCA14 as follows: lane 9, tongue; 10, brain (cerebellum); 11, thymus; 12, bladder; 13, bone marrow; 14, left ovary; 15, left oviduct. A GPI1AB heteropolymer band is seen in the sample of tongue in lane 9 because cell fusion occurs in skeletal muscle. Lane 16 is a control blood sample from a heterozygous *Gpi1*$^{a/b}$ mouse, which also produces a GPI1AB heteropolymer band. Direction of migration (vertical arrows) is from positive to negative. *Abbreviations*: AA, GPI1AA homodimer (GPI1A allozyme band); AB, GPI1AB heterodimer (GPI1AB allozyme band); BB, GPI1BB homodimer (GPI1B allozyme band). **b.** Part of a 3µm thick, toluidine blue-stained plastic section of the left eye from adult *Tg/Tg, Gpi1*$^{b/b}$*, Tyr*$^{+/+}$↔WT, *Gpi1*$^{a/a}$*, Tyr*$^{c/c}$ chimaera AdCA20. The RPE in the region shown is pigmented (*Tg/Tg, Gpi1*$^{b/b}$*, Tyr*$^{+/+}$ cells) to the left of the arrow but albino (WT *Gpi1*$^{a/a}$*, Tyr*$^{c/c}$ cells) to the right. (There is a blood vessel in the thicker pigmented choroid above the RPE.) Scale bar, 20µm. *Abbreviations*: INL, inner nuclear layer, ONL, outer nuclear layer; RPE, retinal pigment epithelium. **c.** Comparisons of composition of different tissues for the three groups of fetal chimaeras shown as mean ± 95% confidence intervals for % pigment RPE of left eyes and % GPI1B for all other samples. Only data for the left eyes were included in the analysis because the % pigment in the left and right eyes were closely correlated (Spearman $r_s = 0.959$; $P < 0.0001$ for the three groups combined). *Abbreviations*: Eye, left eye; F, fetus; Am, amnion; YSM, yolk sac mesoderm; YSE, yolk sac endoderm; PE, parietal endoderm; Tr, trophoblast; Pl, placenta. **d.** Comparisons of composition (% pigment for coat and left eyes and % GPI1B for all other samples) of different tissues for the three groups of adult chimaeras. For clarity, error bars are not shown. (N=2-3 WT↔WT, 14-17 *Tg/-*↔ WT and 6-7 *Tg/Tg*↔WT, excluding sex-specific tissues.) *Abbreviations*: Ct, coat pigment (subjective estimate); Ey, eye pigment (subjective estimate); Br, brain (cerebrum); Bl, blood; Sp, spleen; Ki, left kidney; Mu, left hind limb muscle; To, tongue; H, heart; Fa, left mammary fat pad; St, stomach; SI, small intestine (middle third); LI, large intestine; Lv, liver (medial lobe); Lu, lung; Pa, pancreas; UB, urinary bladder; Gl, sub-maxillary and parotid glands; Te, left testis; Ep, left epididymis; SV, left seminal vesicle; Ov, left ovary; Od, left oviduct; Ut, left uterine horn.

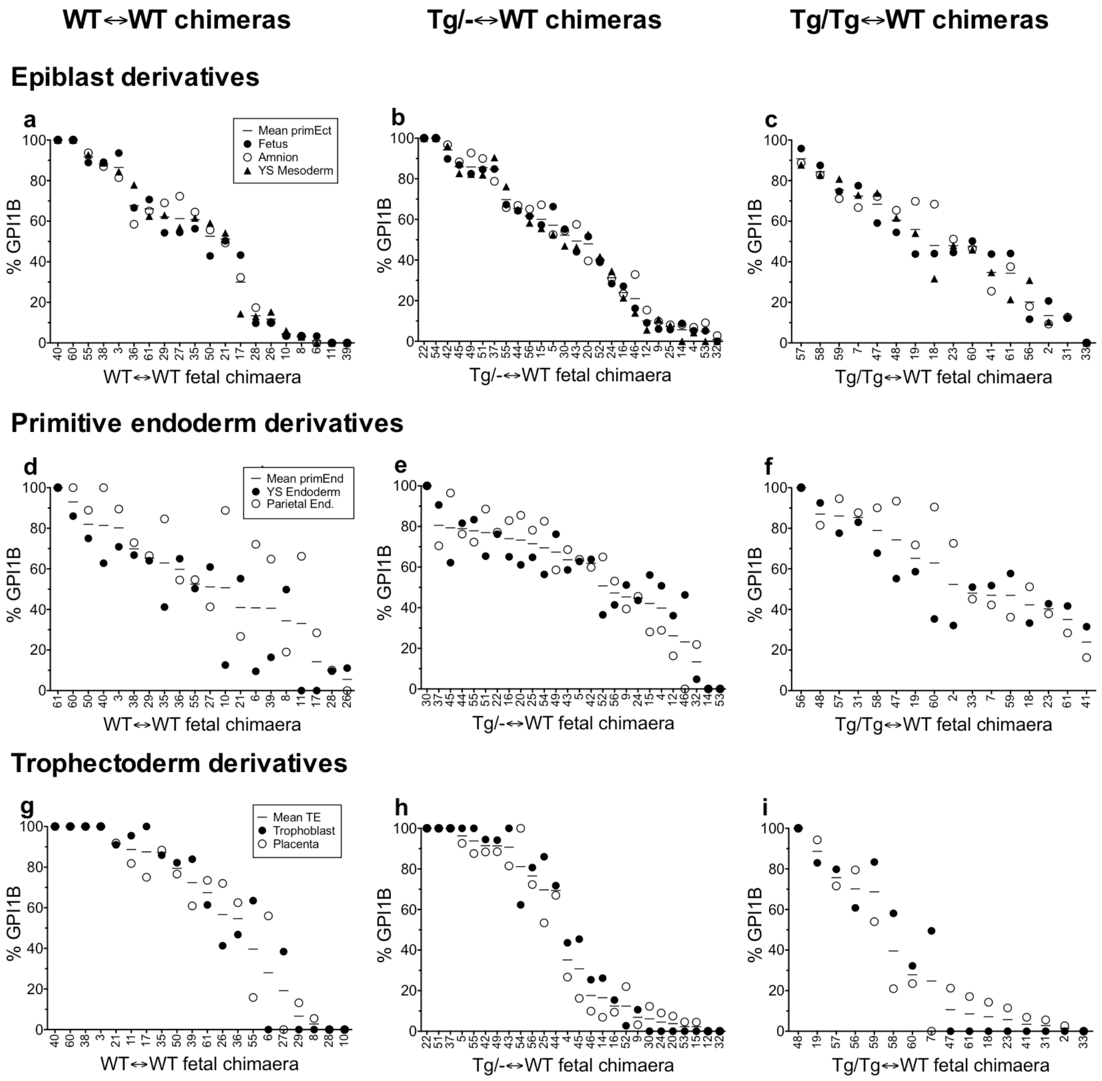

Online Resource 3 (Supplementary Fig. S2). Relationships among individual tissues within each of the three primary developmental lineages in individual E12.5 fetal chimaeras.
Plots of %GPI1B in samples from E12.5 fetal chimaeras for (**a-c**) three epiblast derivatives (fetus, amnion and yolk sac mesoderm) and their mean; (**d-f**) two primitive endoderm derivatives (yolk sac endoderm and parietal endoderm) and their mean; (**g-i**) two samples predominantly of trophectoderm origin (placenta and a trophoblast sample from Reichert's membrane) and their mean. Results are shown separately for (a,d,g) 20 WT (GPI1B)↔WT (GPI1A) chimaeras, (b,e,h) 26 *Tg/-* (GPI1B)↔WT (GPI1A) chimaeras and (c,f,i) 16 *Tg/Tg* (GPI1B)↔WT (GPI1A) chimaeras. In each case the chimaera samples are ordered with the chimaera with the highest %GPI1B on the left of the X-axis to show the relationships among tissues within each of the three primary lineages more clearly. Numbers on X-axis are chimaera identity numbers for chimaeras in series CA.

Online Resource 4 (Supplementary Table S2). Correlation matrices showing Spearman correlation coefficients (r_s) for % GPI1B in fetus and extraembryonic tissues of E12.5 fetal chimaeras

A. WT *Gpi1$^{b/b}$*↔WT *Gpi1$^{a/a}$* chimaeras (N=20)

Tissues compared	Epiblast tissues			Primitive end tissues		Trophectoderm tissues	
	Fetus	Amnion	YS mes	YS end	Par end	Troph	Placenta
Fetus	NA						
Amnion	0.963***	NA					
YS mes	0.967***	0.964***	NA				
YS end	0.728***	0.720***	0.754***	NA			
Par end	0.501*	0.474*	0.487*	0.582**	NA		
Troph	0.522*	0.450*	0.459*	0.259 (NS)	0.398 (NS)	NA	
Placenta	0.543*	0.432 (NS)	0.500*	0.391 (NS)	0.506*	0.896***	NA

B. *Tg/- Gpi1$^{b/b}$*↔WT *Gpi1$^{a/a}$* chimaeras (N=26)

Tissues compared	Epiblast tissues			Primitive end tissues		Trophectoderm tissues	
	Fetus	Amnion	YS mes	YS end	Par end	Troph	Placenta
Fetus	NA						
Amnion	0.967***	NA					
YS mes	0.980***	0.966***	NA				
YS end	0.626***	0.583**	0.659***	NA			
Par end	0.563**	0.487*	0.587**	0.735***	NA		
Troph	0.604**	0.549**	0.584**	0.564**	0.333 (NS)	NA	
Placenta	0.765***	0.721***	0.759***	0.631***	0.500**	0.878***	NA

C. *Tg/Tg Gpi1$^{b/b}$*↔WT *Gpi1$^{a/a}$* chimaeras (N=16)

Tissues compared	Epiblast tissues			Primitive end tissues		Trophectoderm tissues	
	Fetus	Amnion	YS mes	YS end	Par end	Troph	Placenta
Fetus	NA						
Amnion	0.846***	NA					
YS mes	0.908***	0.926***	NA				
YS end	0.168 (NS)	0.282 (NS)	0.365 (NS)	NA			
Par end	0.113 (NS)	0.215 (NS)	0.212 (NS)	0.574*	NA		
Troph	0.446 (NS)	0.503*	0.620*	0.655**	0.283 (NS)	NA	
Placenta	0.296 (NS)	0.508*	0.499*	0.561*	0.446 (NS)	0.755***	NA

Abbreviations: YS mes, Yolk Sac mesoderm; YS end, Yolk Sac endoderm; Par end, parietal endoderm; Troph, Trophoblast NA, not applicable; NS, not significant. *$P < 0.05$; **$P < 0.01$; ***$P < 0.001$.

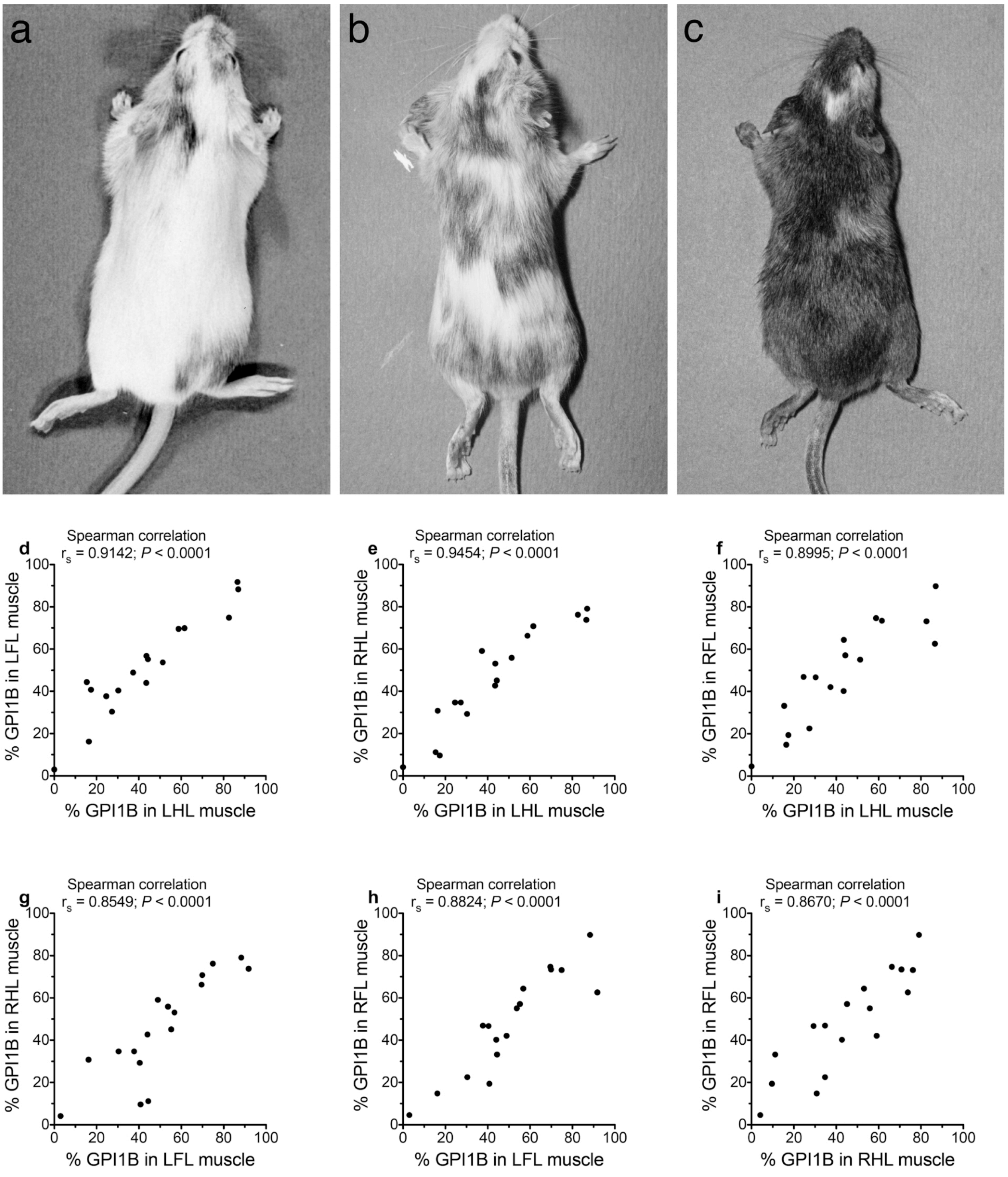

Online Resource 5 (Supplementary Fig. S3). Relationships in composition of samples from left and right side of bodies of adult chimaeras.

a-c. Photographs of three adult *Tg/-, Gpi1*$^{b/b}$*, Tyr*$^{+/+}$↔WT*, Gpi1*$^{a/a}$*, Tyr*$^{c/c}$ chimaeras showing similar levels of pigment in left and right sides of the dorsal coats: (a) male chimaera AdCA8, (b) female AdCA26 and (c) male AdCA19. **d-i** Pairwise comparisons of composition (%GPI1B) of the four skeletal muscles samples from left and right forelimbs and hindlimbs of 17 *Tg/-, Gpi1*$^{b/b}$*, Tyr*$^{+/+}$↔WT*, Gpi1*$^{a/a}$*, Tyr*$^{c/c}$ chimaeras, showing correlations between samples from the same side of the body (d,i) are no stronger than those between different sides (e,f,g,h). Spearman correlation coefficients (r_S) and *P*-values are shown in the figures. Abbreviations: LFL, left forelimb; LHL, left hindlimb; RFL, right forelimb; RHL, right hindlimb.
