# Supplementary material for: Lessons from mouse chimaera experiments with a reiterated transgene marker: revised marker criteria and a review of chimaera markers
Source: Transgenic Res. 2015 Jun 6;24(4):665–91. doi: 10.1007/s11248-015-9883-7 (PMC4504987; doi:10.1007/s11248-015-9883-7)
Supplement: Supplementary file 4 — Online Resource 6 (PDF 138 kb) [file 11248_2015_9883_MOESM4_ESM.pdf]

Online Resource 6 (Supplementary Table S3a, b). Correlation matrices showing Spearman correlation coefficients (r_s) for % GPI1B (or % pigment) in representative tissues in adult *Tg/-* ↔ WT and *Tg/Tg* ↔ WT chimaeras

Supplementary Table S3a: *Tg/- Gpi1$^{b/b}$ Tyr$^{+/+}$* ↔ WT *Gpi1$^{a/a}$ Tyr$^{c/c}$* chimaeras

	Ct	Ey	Br	Bl	Sp	Ki	Mu	To	H	Fa	St	SI	LI	Lv	Lu	Pa	UB	Gl	Te	Ep	SV	Ov	Od	Ut
Number	17	14	17	17	17	17	17	17	17	17	17	17	17	17	17	17	17	15	14	14	14	3	3	3
Ct	NA																							
Ey	0.83***	NA																						
Br	0.90***	0.87***	NA																					
Bl	0.84***	0.79***	0.82***	NA																				
Sp	0.88***	0.89***	0.93***	0.94***	NA																			
Ki	0.84***	0.88***	0.95***	0.81***	0.88***	NA																		
Mu	0.75***	0.82***	0.85***	0.77***	0.82***	0.84***	NA																	
To	0.86***	0.90***	0.89***	0.79***	0.84***	0.84***	0.93***	NA																
H	0.89***	0.87***	0.90***	0.84***	0.88***	0.88***	0.86***	0.92***	NA															
Fa	0.90***	0.86***	0.97***	0.78***	0.87***	0.93***	0.85***	0.91***	0.90***	NA														
St	0.80***	0.78**	0.86***	0.77***	0.86***	0.79***	0.89***	0.94***	0.94***	0.86***	NA													
SI	0.90***	0.93***	0.90***	0.86***	0.93***	0.87***	0.86***	0.89***	0.88***	0.87***	0.83***	NA												
LI	0.77***	0.80***	0.91***	0.74***	0.90***	0.88***	0.88***	0.84***	0.85***	0.87***	0.89***	0.88***	NA											
Lv	0.79***	0.80***	0.87***	0.76***	0.85***	0.86***	0.84***	0.87***	0.90***	0.88***	0.91***	0.84***	0.90***	NA										
Lu	0.87***	0.93***	0.92***	0.91***	0.97***	0.91***	0.81***	0.85***	0.88***	0.88***	0.81***	0.94***	0.87***	0.81***	NA									
Pa	0.79***	0.90***	0.79***	0.81***	0.84***	0.72**	0.81***	0.83***	0.76***	0.74***	0.76***	0.84***	0.76***	0.73***	0.80***	NA								
UB	0.88***	0.81***	0.95***	0.74***	0.85***	0.90***	0.87***	0.89***	0.90***	0.95***	0.87***	0.88***	0.91***	0.85***	0.85***	0.76***	NA							
Gl	0.82***	0.90***	0.93***	0.86***	0.87***	0.90***	0.88***	0.88***	0.86***	0.92***	0.80***	0.88***	0.85***	0.78***	0.88***	0.81***	0.94***	NA						
Te	0.84***	0.71*	0.66*	0.67**	0.69**	0.72**	0.58*	0.75**	0.86***	0.71**	0.68**	0.77**	0.64*	0.73**	0.73**	0.65*	0.75**	0.55	NA					
Ep	0.92***	0.75**	0.75**	0.75**	0.76**	0.78**	0.61*	0.78***	0.85***	0.78***	0.67**	0.83***	0.64*	0.75**	0.76**	0.72**	0.79***	0.62*	0.96***	NA				
SV	0.90***	0.82**	0.99***	0.76**	0.92***	0.93***	0.85***	0.86***	0.86***	0.97***	0.84***	0.89***	0.89***	0.82***	0.89***	0.75**	0.97***	0.93***	0.64*	0.74**	NA			
Ov	1.00		1.00***	0.50	0.50	1.00***	1.00***	1.00***	1.00***	1.00***	1.00***	1.00***	1.00***	1.00***	0.50	1.00***	1.00***	1.00***	NA	NA	NA	NA		
Od	1.00		1.00***	0.50	0.50	1.00***	1.00***	1.00***	1.00***	1.00***	1.00***	1.00***	1.00***	1.00***	0.50	1.00***	1.00***	1.00***	NA	NA	NA	1.00***	NA	
Ut	0.50		0.50	1.00***	1.00***	0.50	0.50	0.50	0.50	0.50	0.50	0.50	0.50	0.50	1.00***	0.50	0.50	0.50	NA	NA	NA	0.50	0.50	NA

Abbreviations: Ct, coat pigment (subjective estimate); Ey, eye pigment (subjective estimate); Br, brain (cerebrum); Bl; blood, Sp, spleen; Ki, left kidney; Mu, left hind limb muscle; To, tongue; H, heart; Fa, left mammary fat pad; St, stomach; SI, small intestine (middle third); LI, large intestine; Lv, liver (medial lobe), Lu, lung; Pa, pancreas; UB, urinary bladder; Gl, sub-maxillary and parotid glands; Te, left testis, Ep, left epididymis; SV, left seminal vesicle; Ov, left ovary; Od, left oviduct; Ut, left uterine horn; NA, not applicable; * $P<0.05$; ** $P<0.01$; *** $P<0.001$.

Supplementary Table S3b: *Tg/Tg Gpi1$^{b/b}$ Tyr$^{+/+}$* ↔ WT *Gpi1$^{a/a}$ Tyr$^{c/c}$* chimaeras

	Ct	Ey	Br	Bl	Sp	Ki	Mu	To	H	Fa	St	SI	LI	Lv	Lu	Pa	UB	Gl	Te	Ep	SV	Ov	Od	Ut
Number	7	7	7	7	7	7	7	7	7	6	6	7	7	7	7	6	7	6	5	5	5	1	1	2
Ct	NA																							
Ey	0.52	NA																						
Br	0.93**	0.63	NA																					
Bl	0.54	-0.09	0.39	NA																				
Sp	0.43	0.77*	0.64	0.14	NA																			
Ki	0.75	0.70	0.93**	0.32	0.82*	NA																		
Mu	0.63	0.66	0.83*	-0.14	0.68	0.83*	NA																	
To	0.43	0.23	0.50	-0.32	0.14	0.39	0.74	NA																
H	0.79*	0.61	0.93**	0.25	0.57	0.93**	0.81*	0.50	NA															
Fa	0.83	0.75	0.94*	0.37	0.71	0.89*	0.64	-0.09	0.83	NA														
St	0.37	0.71	0.71	0.03	0.89*	0.94*	0.75	0.60	0.83	0.90	NA													
SI	0.79*	0.58	0.89*	0.29	0.61	0.82*	0.77*	0.29	0.82*	0.94*	0.66	NA												
LI	0.71	0.70	0.89*	0.36	0.86*	0.96**	0.77*	0.21	0.86*	0.94*	0.94*	0.89*	NA											
Lv	0.75	0.52	0.89*	0.07	0.46	0.82*	0.88*	0.61	0.93**	0.77	0.66	0.89*	0.79*	NA										
Lu	0.75	0.70	0.93**	0.32	0.82*	1.00**	0.83*	0.39	0.93**	0.89*	0.94*	0.82*	0.96**	0.82*	NA									
Pa	0.54	0.75	0.60	0.09	0.94*	0.71	0.89*	0.26	0.54	0.70	0.70	0.66	0.77	0.60	0.71	NA								
UB	0.75	0.41	0.86*	0.29	0.36	0.82*	0.70	0.54	0.96**	0.66	0.83	0.71	0.71	0.89*	0.82*	0.26	NA							
Gl	0.77	0.20	0.89*	0.83	0.31	0.83	0.54	0.26	0.94*	0.70	0.70	0.77	0.71	0.89*	0.83	0.26	1.00**	NA						
Te	0.10	1.00*	0.00	-0.30	0.70	0.20	0.30	0.40	0.10	0.20	0.50	0.00	0.20	0.00	0.20	0.60	0.10	0.10	NA					
Ep	0.10	0.70	0.30	0.00	1.00*	0.70	0.70	0.50	0.40	0.40	0.90	0.30	0.70	0.30	0.70	0.90	0.40	0.40	0.70	NA				
SV	0.70	0.10	0.90	0.90	0.40	0.90	0.50	0.30	1.00*	1.00	0.70	0.90	0.90	0.90	0.90	0.30	1.00	1.00*	0.10	0.40	NA			
Ov																			NA	NA	NA	NA		
Od																			NA	NA	NA		NA	
Ut																			NA	NA	NA			NA

Abbreviations: Ct, coat pigment (subjective estimate); Ey, eye pigment (subjective estimate); Br, brain (cerebrum); Bl; blood, Sp, spleen; Ki, left kidney; Mu, left hind limb muscle; To, tongue; H, heart; Fa, left mammary fat pad; St, stomach; SI, small intestine (middle third); LI, large intestine; Lv, liver (medial lobe), Lu, lung; Pa, pancreas; UB, urinary bladder; Gl, sub-maxillary and parotid glands; Te, left testis, Ep, left epididymis; SV, left seminal vesicle; Ov, left ovary; Od, left oviduct; Ut, left uterine horn; NA, not applicable; * P <0.05; ** P <0.01.
