# Supplementary material for: Lessons from mouse chimaera experiments with a reiterated transgene marker: revised marker criteria and a review of chimaera markers
Source: Transgenic Res. 2015 Jun 6;24(4):665–91. doi: 10.1007/s11248-015-9883-7 (PMC4504987; doi:10.1007/s11248-015-9883-7)
Supplement: Supplementary file 5 — Online Resource 7 & 8 (PDF 196 kb) [file 11248_2015_9883_MOESM5_ESM.pdf]

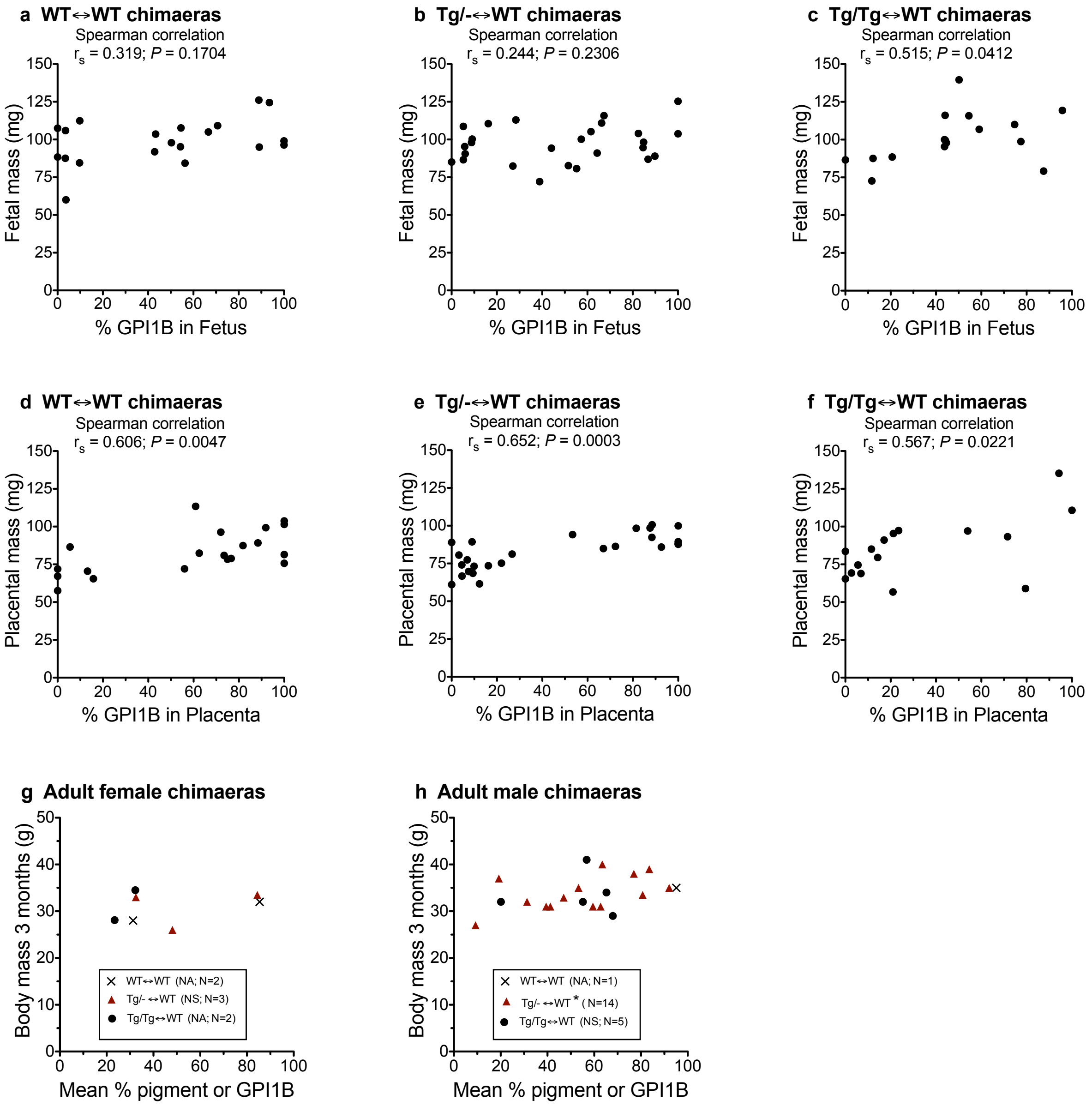

Online Resource 7 (Supplementary Fig. S4). Relationships between mass and composition of WT↔WT, *Tg/-*↔WT and *Tg/Tg*↔WT chimaeras

a-c. Associations between fetal mass and %GPI1B in the fetus for (a) 20 WT (GPI1B) ↔WT (GPI1A) chimaeras, (b) 26 *Tg/-* (GPI1B) ↔ WT (GPI1A) chimaeras and (c) 16 *Tg/Tg* (GPI1B)↔WT (GPI1A) chimaeras. Fetal mass was significantly positively correlated with % fetal GPI1B for only the *Tg/Tg*↔WT chimaeras, as shown in the figures. **d-f.** Associations between placental mass and %GPI1B in the placenta for the same three groups of chimaeras. Placental mass was significantly positively correlated with % placental GPI1B for each of the genotype combinations, as shown in the figures. **g,h.** Relationships between 3-month body mass and mean composition of the tissues (mean of the % pigment or % GPI1B in 21 tissues) in (g) female chimaeras and (h) male chimaeras. There were only sufficient pairs for a valid analysis for *Tg/-*↔WT and *Tg/Tg*↔WT male chimaeras. Only the male *Tg/-*↔WT chimaeras showed a significant correlation between composition and body mass (Spearman $r_s = 0.5473$; $P = 0.0428$) and this was entirely dependent on the smallest mouse.

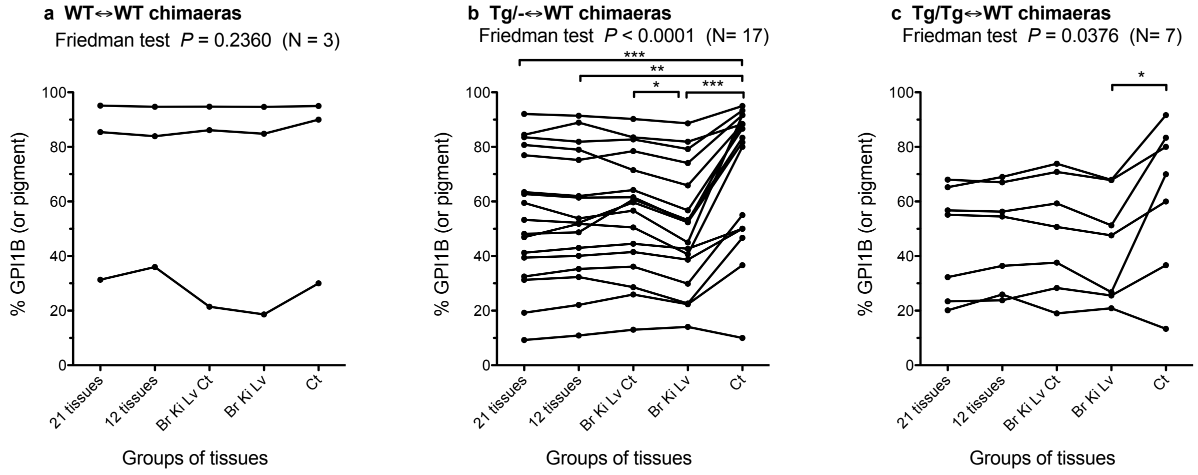

Online Resource 8 (Supplementary Fig. S5). Comparisons of compositions of different combinations of tissue samples from WT↔WT, *Tg/-*↔WT and *Tg/Tg*↔WT adult chimaeras
Comparison of the mean composition (% GPI1B or pigment) of 21 tissues with the mean of 12 tissues, four tissues (brain, kidney, liver and coat pigment), three tissues (brain, kidney and liver) or one tissue (coat pigment) in individual adult chimaeras: (**a**) WT↔WT, (**b**) *Tg/-*↔WT and (**c**) *Tg/Tg*↔WT. Abbreviations: Ct, coat pigment (subjective estimate); Br, brain (cerebrum); Ki, left kidney; Lv, liver (medial lobe). See text for explanations of 21 and 12 tissues respectively. Significance values for Dunn's post-hoc tests: * $P < 0.05$; ***$P < 0.001$.
